# Characterization and Exploration of the Neuroprotective Potential of Oat-Protein-Derived Peptides in PC12 Cells and Scopolamine-Treated Zebrafish

**DOI:** 10.3390/nu16010117

**Published:** 2023-12-29

**Authors:** Hamad Rafique, Xinzhong Hu, Tian Ren, Rui Dong, Rana Muhammad Aadil, Liang Zou, Mian Kamran Sharif, Lu Li

**Affiliations:** 1 College of Food Engineering and Nutritional Science, Shaanxi Normal University, Xi’an 710119, China; hamad2020@snnu.edu.cn (H.R.);; 2 National Institute of Food Science and Technology, University of Agriculture, Faisalabad 38000, Pakistan; 3 School of Food and Biological Engineering, Chengdu University, Chengdu 610106, China; 4 Guilin Seamild Food Co., Ltd., Guilin 541000, China

**Keywords:** oat peptides, biological activity, PC12 cells, oxidative stress, neuroprotection, zebrafish, Nrf2-keap1, Bdnf

## Abstract

Neurodegenerative disorders pose a substantial risk to human health, and oxidative stress, cholinergic dysfunction, and inflammation are the major contributors. The purpose of this study was to explore the neuroprotective effects of oat protein hydrolysate (OPH) and identify peptides with neuroprotective potential. This study is the first to isolate and identify OPH peptides with neuroprotective potential, including DFVADHPFLF (DF-10), HGQNFPIL (HL-8), and RDFPITWPW (RW-9), by screening via peptidomes and molecular-docking simulations. These peptides showed positive effects on the activity of antioxidant enzymes and thus reduced oxidative stress through regulation of Nrf2-keap1/HO-1 gene expression in vitro and in vivo. The peptides also significantly ameliorated scopolamine-induced cognitive impairment in the zebrafish model. This improvement was correlated with mitigation of MDA levels, AChE activity, and levels of inflammatory cytokines in the brains of zebrafish. Furthermore, these peptides significantly upregulated the mRNA expression of Bdnf, Nrf2, and Erg1 in the brains of zebrafish with neurodegenerative disorders. Collectively, oat peptides have potential for use as active components in nutraceutical applications for the prevention of neurodegenerative diseases.

## 1. Introduction

Neurodegenerative diseases such as Parkinson’s disease, Alzheimer’s disease (AD), and multiple sclerosis exert substantial societal and familial burdens. These conditions are characterized by several detrimental factors, including dysfunction of the cholinergic system, heightened oxidative stress, inflammatory responses, the presence of neurofibrillary tangles, and the formation of amyloid plaques. The proper functioning of cholinergic neurons and their associated neurotransmitters and receptors is crucial for effective signal transmission within the central nervous system. Disruptions in this process impair neural signaling, leading to diminished learning and memory capacity [1]. Additionally, uncontrolled oxidative stress during the progression of neurodegenerative disorders contributes to an imbalance in the cholinergic system [2]. For instance, studies have demonstrated that lipid peroxidation contributes to an increase in acetylcholinesterase activity [3]. The close connection between oxidative stress and the inflammatory response is also widely recognized. Excessive production of free radicals through oxidative stress indirectly promotes inflammation and accelerates the progression of neurodegeneration [4,5,6]. The activation of inflammatory genes by ROS leads to the overproduction of pro-inflammatory mediators, contributing to the initiation and progression of neuroinflammation [7] and subsequently enhancing ROS production. Neurodegeneration is intimately correlated with a compromised nuclear factor (Nrf2) defensive pathway [8]. Under conditions of oxidative stress, ROS or inhibitors of the Keap1–Nrf2 interaction can stimulate the release of Nrf2, promoting its nuclear translocation and binding to the antioxidant response element (ARE) and thereby increasing the expression of cytoprotective genes [9]. Nrf2 also regulates various cytoprotective proteins, including anti-inflammatory, anti-apoptotic, and neurotrophin proteins [9,10]. This connection highlights the importance of oxidative stress, the cholinergic system, and inflammation in the progression of cognitive impairments. Natural substances with antioxidant properties have potential for use in treating neurodegenerative disorders, and their use has increased compared to synthetic antioxidants owing to safety concerns [11,12,13,14]. Peptides derived from dietary protein sourced from animals, marine life, insects, and plants, are highly favorable in terms of digestion, absorption, excretion, and lack of adverse effects [15,16,17,18,19]. Generally, hydrolysates or peptides with bioactive properties are obtained by the enzymatic hydrolysis of proteins. Ultrasonic processing has gained attention as an eco-friendly, cost-effective, and safe method for extracting natural substances [20]. The use of ultrasonics enhances the preparation and activity of bioactive peptides by altering protein structure and exposing cleavage sites, enabling the efficient development of protein resources and facilitating the production of bioactive peptides [21]. Thus, ultrasound-assisted enzymolysis is a suitable approach for the preparation of bioactive short-chain peptides.

Oats are regarded as a highly nutritious grain because they are rich in phytochemicals, dietary fibers, and protein. Oat proteins and peptides have recently emerged as promising candidates for therapeutic and nutraceutical applications [22]. Oat peptides have been extensively studied for antidiabetic, antihypertensive, antioxidant, anticholesterolemic, and immunomodulatory properties, among others [22]. However, the neuroprotective effects of oat proteins and peptides remain relatively unexplored. Therefore, this study aimed to investigate the neuroprotective effects of ultrasound-assisted enzymatically hydrolyzed OPH in PC12 cells subjected to oxidative stress. Peptidomics approaches, along with in silico predictions employing Peptide Ranker and molecular docking, were utilized to identify neuroprotective peptides. The peptides (DF-10, HL-8, and RW-9) were further evaluated for their potential to mitigate memory impairment by modulating AChE activity, oxidative stress, and inflammation in scopolamine-exposed zebrafish. Additionally, mRNA expression levels of brain-derived neurotropic factor (Bdnf), Nrf2 antioxidant defense-related protein, and Erg1 neural activity-related protein were measured to investigate the mechanism of action of oat peptides. 

## 2. Materials and Methods 

### 2.1. Materials

Oat bran flour was provided by Guilin Seamild Food Co., Ltd., Guilin, China. The composition of the oat bran was as follows: starch, 49.32%; protein, 17.90%; lipids, 8.13%; and, β-glucan 6.43%. PC-12 cells (Procell CL-0481) were purchased from Procell Life Science & Technology (Wuhan, China). RPMI-1640 and penicillin-streptomycin were purchased from Hyclone (Logan, UT, USA); trypsin-EDTA solution was purchased from Biosharp Life Science (Hefei, China); and DMSO was purchased from Sigma (St. Louis, MO, USA). ROS and superoxide dismutase (SOD) assay kits were purchased from Beijing Solarbio Science & Technology (Beijing, China). Lactate dehydrogenase (LDH), catalase (CAT), and glutathione (GSH-px) assay kits were purchased from Nanjing Jiancheng Bioengineering Institute (Nanjing, China). Scopolamine and piracetam were purchased from Shanghai Macklin Biochemical Technology Co., Ltd., Shanghai, China. AchE, MDA, and GSH assay kits were purchased from Solarbio Science & Technology Co., Ltd., Beijing, China. The peptides DFVADHPFLF (DF-10), HGQNFPIL (HL-8), and RDFPITWPW (RW-9) with >98% purity were synthesized by Jiangsu Ji Tai Peptide Industry Science and Technology Co. (Yancheng, Jiangsu, China). All the reagents used in this study were of analytical grade. 

### 2.2. Extraction of Oat Protein Isolates (OPI)

The OPI were extracted from oat bran using a modified version of the method reported in [23]. Briefly, defatted oat-bran flour was mixed with distilled water at a ratio of 1:15 (*w*/*v*). Following a pH adjustment to 9.5, the suspension was stirred for 60 min at room temperature. Then, the slurry was centrifuged at 4000 rpm for 20 min to remove the precipitate. The supernatant was collected, adjusted to pH 4.5 using 1.0 M HCl and centrifuged at 4000 rpm for 15 min to precipitate proteins. The extracted proteins were removed and stored in plastic bags at −20 °C for further study. The protein content of the OPI was 86.3%, as determined by the Kjeldahl method.

### 2.3. Ultrasound-Pretreated Enzymatic Hydrolysis of Oat Protein Isolates

OPI suspensions were treated with ultrasound waves with the help of a probe (Φ6) at 25 Hz with different levels of ultrasonic power (200 W, 400 W, and 600 W) for 15 min using an ultrasound instrument (Xinyi 950-N, Ningbo Xinyi Ultrasonic Instrument Co., Ltd., Ningbo, China). After the pH was adjusted to 8, samples were placed in an incubator at 55 °C. Next, alcalase 10,000 U/g 2% (*w*/*w*) was added and the samples were continuously shaken at 150 rpm for 1 h to hydrolyze the proteins. The hydrolysates were then centrifuged, and the supernatant was lyophilized to obtain oat hydrolysate powder, which was stored at −20 °C for further study.

### 2.4. Estimating the Degree of Hydrolysis (DH) and In Vitro Antioxidant Capacity of OPH

The ninhydrin colorimetric method was used to determine the degree of protein hydrolysis. In brief, 1 mL of hydrolysis supernatant was transferred to a volumetric flask, adjusted to pH 6.0, and diluted with distilled water to 100 mL. Then, 2 mL of diluent was transferred into a test tube, and 1 mL of ninhydrin solution was added, mixed well, and heated in boiling water for 15 min. After cooling to room temperature, 5 mL of ethanol was added, and the mixture was allowed to stand for 15 min. Finally, a standard curve was used to determine the DH from the absorbance recorded at 570 nm. Additionally, the DPPH-radical scavenging capacity was determined by using the previously described method [24], while the hydroxyl-radical scavenging and iron-chelating capacities of the OPH were analyzed according to the method described in [25], with some modifications. 

### 2.5. Cell Culturing and Treatments

PC12 rat adrenal pheochromocytoma cells were cultured in RPMI 1640 medium containing 10% fetal bovine serum and 1% penicillin at a temperature of 37 °C in an environment containing 5% CO_2_. The cells (10^4^ cells/well) were then plated in a 96-well plate. After 24 h, the cells were first incubated with the samples for another 24 h, then treated with 0.2 mM H_2_O_2_ for an additional 24 h. 

#### 2.5.1. Assessment of Viability and Quantification of Lactate Dehydrogenase 

After treatment, the cells were incubated with CKK-8 for 2–4 h at 37 °C. Next, the absorbance at 450 nm was measured using a microplate reader and viability was calculated. Following cell treatment, media were collected from the 24-well plate and the supernatant was utilized for the LDH-release test per the manufacturer’s instructions. The absorbance was recorded at 450 nm using a microplate reader, and the results were expressed as a percentage of the control group.

#### 2.5.2. Nuclear-Morphology Analysis

Cells were treated and then washed twice with PBS. Next, the cells were stabilized by exposure to a 4% paraformaldehyde solution at room temperature for a period of twenty minutes. The cells were then treated with Hoechst 33,258 (10 µg/mL) and placed in the dark at ambient temperature for 20 min. The cells were then rinsed, and an inverted microscope was used to observe them [26].

#### 2.5.3. Measurement of ROS Concentration

The DCFH-DA protocol test was used to quantify the levels of intracellular ROS. Briefly, cells were treated with DCFH-DA for 30 min at 37 °C in the absence of light, then washed three times with growth medium and once with PBS. In order to measure DCF fluorescence, a microplate reader was used with excitation at 488 nm and emission at 530 nm.

#### 2.5.4. Assessing the Activity of CAT, SOD and GSH-px

After the treatments described above, the cells were collected, washed, and lysed. The lysed cells were centrifuged at a speed of 13,000× *g* for ten minutes. SOD, GSH-px, and CAT activity were measured from the collected supernatant using the appropriate assay kits.

### 2.6. RNA Isolation and Quantification by RT-qPCR

After appropriate treatment, total RNA was extracted from the cells using Trizol reagent. The forward and reverse primers used were those given in (Supplementary Table S1). Real-time PCR was performed with newly designed primers, following the standard protocol.

### 2.7. Identification of Peptides 

Liquid A contained 0.1% formic acid in water, and liquid B contained 0.1% formic acid in 84% acetonitrile. A liquid chromatographic column (0.15 mm × 150 mm, RP-C18, Column Technology Inc., Fremont, CA, USA) was equilibrated with 95% Liquid A. The sample was loaded from an autosampler into Zorbax 300SB-C18 peptide traps (Agilent Technologies, Wilmington, DE, USA) and re-processed for separation using a liquid chromatography column. The linear gradient of Liquid B was 4–50%, 50–100%, then 100%, maintained for 0–50 min, 50–54 min, and 54–60 min, respectively. After separation, the peptides were identified by mass spectrometry with a Q-exactive mass spectrometer (Thermo Fisher, Waltham, MA, USA), and the instrument was operated for 60 min with the positive-ion-detection method. The mass-to-charge ratio of peptides was collected as ten fragment profiles (MS2 scan) after a full scan each time. Proteomic software (MaxQuant 1.5.5.1) was used to search the corresponding database for raw files from the mass-spectrometry test. Finally, the proteins were identified. MS/MS data searches were conducted in the National Center for Biotechnology Information (NCBI) database. For identification and quantitative analysis, the parameters and descriptions used in the database search were as follows: variable modification, oxidation (M); max missed cleavage, 2; decoy database pattern, reverse; peptide tolerance, 20 ppm; ms/ms tolerance, 0.1 Da; and FDR, ≤0.01.

### 2.8. Molecular Docking

The binding affinities of peptides to the Keap1 protein were evaluated using molecular docking. The structure of the 6QMJ target protein was downloaded from the RCSB database. After the protein was cleaned and unnecessary small molecules were removed from the protein using the UCSF Chimera program, the protein was imported into AutoDock Tools 1.5.6 software to delete water molecules, add hydrogen atoms, and set atom types. Molecular docking was performed using AutoDock Vina, and the predicted peptide-Keap1 binding affinity was expressed as the Vina binding affinity in kcal/mol. Thus, the lowest possible Vina score indicates the strongest binding affinity. Pymol version 2.5 was used for both the molecular analysis and the visualization.

### 2.9. Animal Housing and Treatments

Zebrafish for the study were obtained from China Zebrafish Resource Centre (CZRC) Wuhan, China. One hundred adult zebrafish (*Danio rerio*), both males and females (50:50), were housed in an aerated water tank for 10 days at a temperature of 26 ± 0.5, a pH of 7 ± 0.1, with dissolved oxygen levels at 6 ± 0.2 mg/L. They were fed twice a day with commercial food. Stock solutions of all three peptides and piracetam (20 and 200 mg/L, respectively) were freshly prepared using distilled water. The zebrafish were allocated into six distinct experimental groups, each consisting of 16 fish. The groups were as follows: the control group, the model group, the RW-9 group, the HL-8 group, the DF-10 group, and the Piracetam group (positive control). All groups were treated with the samples for 14 days, and water was changed every 2 days. Before they were subjected to behavioral assessments, all groups except the control group underwent individual immersion in a 200 µM scopolamine solution for 1 h to induce the zebrafish model of memory impairment.

### 2.10. T-Maze Testing

The zebrafish T-maze test was conducted as per [27], with slight modifications. Zebrafish were initially introduced to a 40 cm-long arm and then made a choice between two 15 cm-long arms, all measuring 15 cm in depth and 6 cm in width. One of the shorter arms led to a reservoir composed of a 20 cm-deep square tank (23 cm × 23 cm) containing artificial grass and stones. Once trained, the majority of zebrafish preferred to spend their time in this reservoir. The fish behaviors were recorded and analyzed using KE-Maze^®^ (Version 3.3.0.0) software (Nanjing Calvin Biotechnology Co., Ltd., Nanjing, China). Recorded parameters included the escape latency (time taken to locate the reservoir), target time (time spent in the reservoir), and distance traveled by the zebrafish.

### 2.11. Determination of Biochemical Parameters in the Zebrafish Brain

After the behavioral assessments, the fish were euthanized through rapid cooling to minimize potential biochemical or physiochemical alterations that could affect postmortem analysis. Afterwards, whole brains were collected for biochemical examination. Subsequently, the brain tissues were subjected to homogenization for 1 min at 1000 rpm in 0.1 M potassium phosphate buffer (pH 7.4) with the K-3-F High Speed Low-Temp. Tissue Homogenizer (Servicebio Technology Co., Ltd., Wuhan, China) equipped with 1 mm-diameter magnetic balls. The homogenate was centrifuged at 14,000 rpm (4 °C) for 15 min, and the resultant supernatant was used to measure the levels of AchE, MDA, GSH, and T-AOC. Levels of inflammatory cytokines, including TNF-α, IL-6, and IL-1β, were also determined.

### 2.12. RNA Isolation and Quantification by RT-qPCR

RT-qPCR was used to measure the mRNA expression levels of nuclear factor erythroid-related factor 2 (Nrf2), brain-derived neurotropic factor (Bdnf), and early-response growth protein-1 (Erg1) in the zebrafish brain. The forward and reverse primers used were those given in (Supplementary Table S1).

### 2.13. Statistical Analysis

Data-processing software (DPS 7.05) was employed to carry out the statistical analysis, and the graphs were constructed using Origin Graphing and Analysis 2015 software. Experiments were repeated three times, and the data were expressed as the mean ± the standard deviation. Differences were considered statistically significant at *p* < 0.05.

## 3. Results and Discussion 

### 3.1. Effect of Ultrasound Pretreatment on the Degree of Hydrolysis (DH) and Antioxidant Activities of OPH

**Figure 1:**
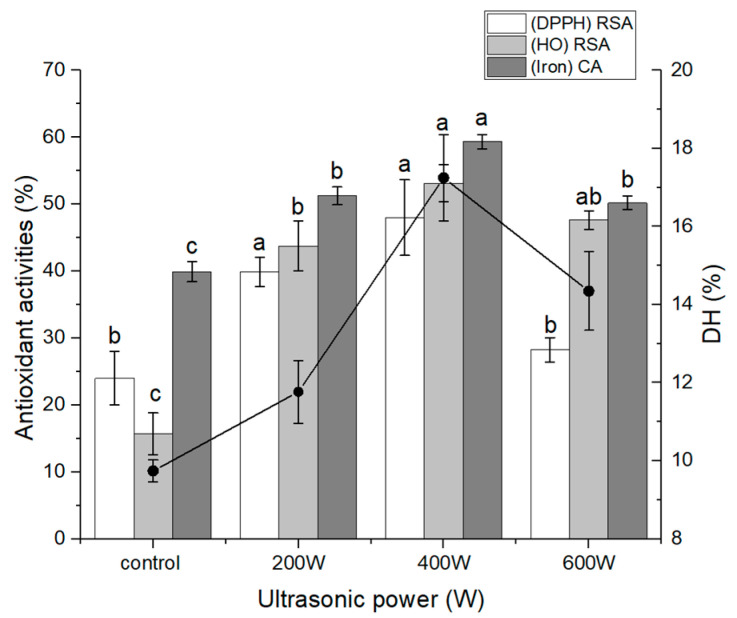
Effect of ultrasound pretreatment on the DH and antioxidant properties of oat protein hydrolysate. The different letters (a, b, c) represent significant differences in DH and activity among groups (*p* < 0.05).

Oxidative stress causes neuronal damage and death, which contribute to the degenerative process of some neuron-related illnesses such as Parkinson’s disease and Alzheimer’s disease [27]. Therefore, antioxidant-targeted drugs have been regarded as having potential for use in the treatment or prevention of neurodegenerative disorders [27]. Bioactive peptides are attracting more and more interest as a potential treatment for neurodegenerative diseases due to their high membrane penetration, biological activities, and ease of absorption in comparison to other types of active compounds [28]. Numerous studies have shown that bioactive peptides can protect neurons by acting as antioxidants and inhibiting ROS, apoptosis, and mitochondrial dysfunction in cell lines and animal models. These benefits can be attributed to the fact that bioactive peptides have been shown to function primarily through these mechanisms [29,30]. In the present study, we prepared antioxidant oat protein hydrolysates using a combined method of multi-intensity ultrasound (powers of 200, 400, and 600 W) followed by enzymatic hydrolysis. It was observed that higher-power ultrasonic treatment significantly improved the DH of oat protein. The DH increased as ultrasonic power increased up to a point, beyond which the DH began to decrease as ultrasonic power increased (Figure 1). This result was quite similar to the results of a previous study that evaluated the effect of ultrasound pretreatment on the physicochemical and bioactive properties of whey protein hydrolysate [31]. Notably, treatment with 400 W ultrasound power resulted in the highest DH value and most effective antioxidant activities. Ultrasound is known to induce various effects, such as cavitation, as well as mechanical and thermal effects, which can alter protein structure by increasing extensibility and exposing more enzyme sites [32]. These effects can improve the efficiency of enzymatic hydrolysis by promoting enzyme-protein interaction. The effectiveness of ultrasound depends on multiple treatment variables, including frequency, waveform, duration, and power. To optimize the mechanical and cavitation effects, appropriate treatment parameters need to be selected. However, excessive power and time can cause protein refolding and curling, potentially re-enclosing exposed enzymes within the protein interior [33]. Appropriate ultrasonic treatment conditions were necessary to enhance DH and antioxidant activities during enzymolysis. Consistent with previous research on oxhide-gelatin-protein hydrolysate and jackfruit-protein isolates, our study also observed increased DH following ultrasonic pretreatment [34,35]. However, in contrast, a different investigation reported no significant improvement in the DH of rice protein with ultrasonication [36]. This variability in results may be attributed to the distinct characteristics of the protein under investigation. Peptides exhibit diverse biological activities that are influenced by factors such as chain length, DH, amino-acid composition, sequence, and conformation [37]. In Figure 1, all tested OPHs demonstrated DPPH, hydroxyl-radical scavenging, and iron-chelating activity. Notably, ultrasound-pretreated hydrolysates displayed higher activity compared to non-treated hydrolysates, with the OPH pre-treated at 400 W exhibiting the highest activity. This result suggests that ultrasonic pretreatment led to the formation of short-chain peptides capable of effectively neutralizing free radicals, thereby interrupting the radical chain reaction and stabilizing the molecules. The ultrasound-pretreated OPH (400 W) with the highest DH and antioxidant activities is the focus of the subsequent investigations in this study.

### 3.2. Neuroprotective Effect of OPH in PC12 Cells

#### 3.2.1. Effect of OPH on Cell Viability, LDH Release, and Nuclear Morphology

**Figure 2:**
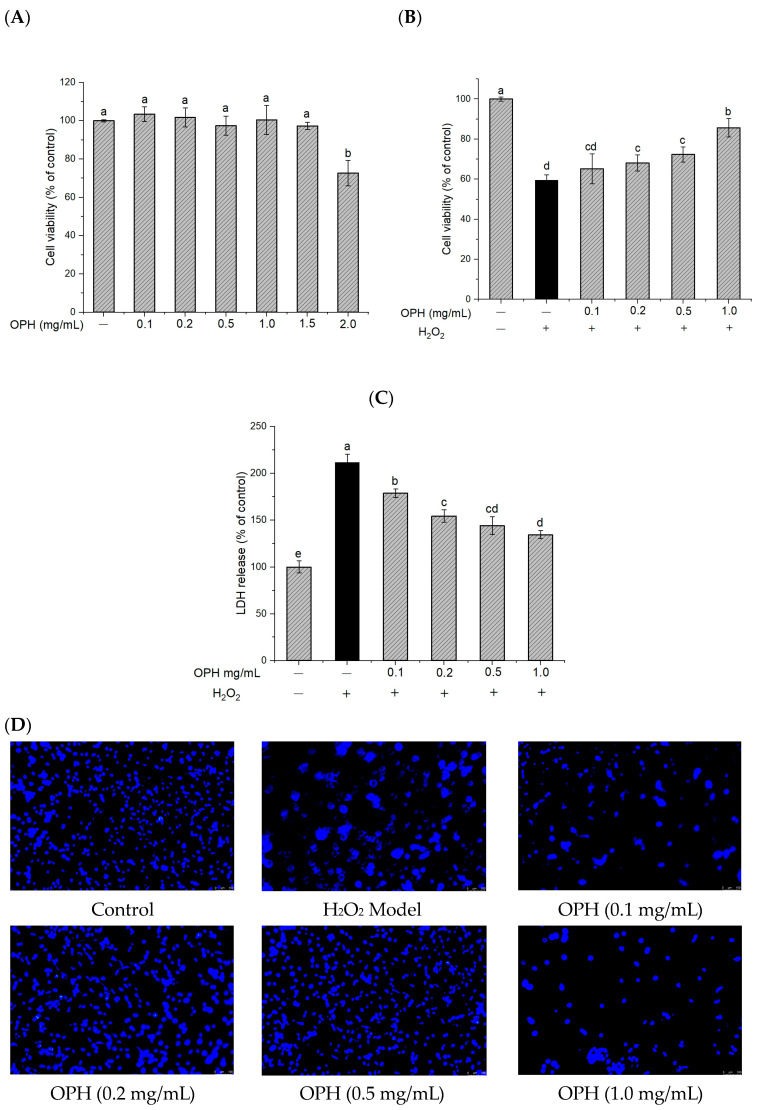
Effect of OPH on cell viability and LDH release in H_2_O_2_ -damaged PC12 cells. (**A**) sample cytotoxicity n = 3; (**B**) cell viability with sample and H_2_O_2_ n = 3; (**C**) LDH release, n = 3; (**D**) Hoechst 33,252 staining under an inverted microscope. Different letters (a, b, c, d, e) represent significant differences among groups (*p* < 0.05). (− and + in the graph represent the absence and presence of OPH or H_2_O_2_ in treatments).

The results of the CKK-8 test demonstrated that OPH (0–1.5 mg/mL) was non-toxic to PC12 cells (Figure 2A). In this study, exposure to 0.2 mM hydrogen peroxide was employed as a model of oxidative stress. At this concentration, the viability of cells was reduced to 57.34 ± 1.05%. The cell viability was significantly increased (*p* < 0.05) by pretreatment with OPH in a dose-dependent manner, with OPH doses ranging from 0.1 to 1.0 mg/mL, before exposure to H_2_O_2_ (Figure 2B). In addition, the release of LDH is a crucial indicator of both cell-membrane integrity and the degree to which the cell has been damaged. After exposure to H_2_O_2_, LDH leakage was found in the culture medium, showing that cell-membrane integrity had been disrupted. The findings of this study revealed that pretreatment with OPH was able to significantly reduce the amount of LDH that was released in response to hydrogen-peroxide exposure (*p* < 0.05) (Figure 2C).

In addition, the morphology of nuclear chromatin was studied as a potential biomarker of apoptosis in cells. After staining, the morphological characteristics of nuclear chromatin were observed. OPH treatment was shown to be partially effective in reversing nuclear condensation, membrane blebbing, nuclear fragmentation, and the formation of apoptotic bodies (Figure 2D).

#### 3.2.2. Effect of OPH on the Inhibition of ROS Generation in H_2_O_2_-Damaged Cells 

**Figure 3:**
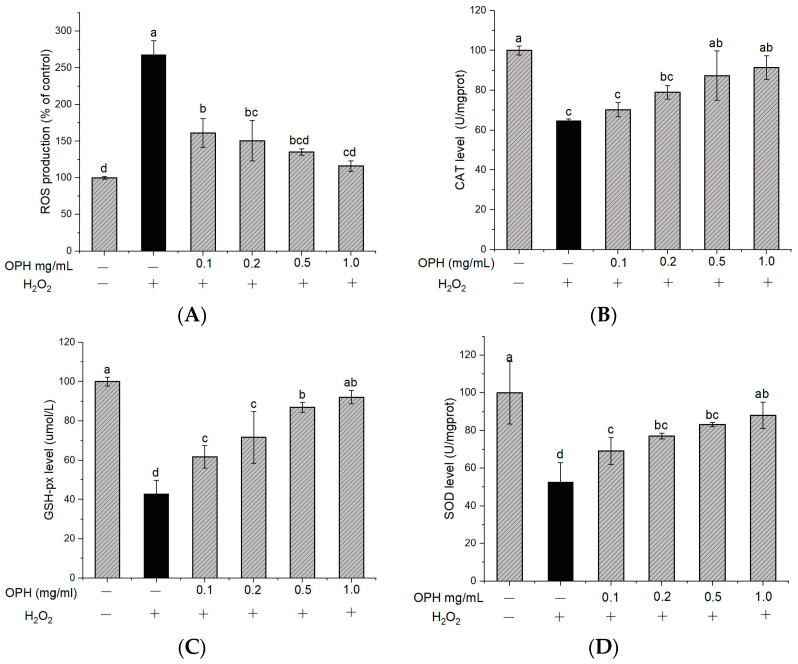
Effect of OPH on the levels of ROS, CAT, GSH-px and SOD in PC12 cells. (**A**) Reactive oxygen species (ROS); (**B**) catalase (CAT) activity, n = 3; (**C**) glutathione (GSH-px), n = 3; (**D**) superoxide dismutase (SOD); n = 3. Different letters (a, b, c, d) represent significant differences among groups (*p* < 0.05). (− and + in the graph represent the absence and presence of OPH or H_2_O_2_ in treatments).

To determine the effect of OPH on the oxidative damage induced by H_2_O_2_, the intracellular ROS level was quantified using fluorescence with DCFH-DA. Figure 3A shows that the accumulation of ROS following exposure to H_2_O_2_ was about 2.6 times higher than that in the control group. In addition, this increase in ROS generation was greatly reduced by OPH pretreatment in a dose-dependent manner (*p* < 0.05). ROS generation is one of the most prominent characteristics of neuronal damage, in particular of H_2_O_2_-induced cell toxicity [38], and ROS accumulation is linked to cell death, which may lead to oxidative damage through lipid peroxidation [39]. A number of studies have shown that antioxidants may prevent or delay the apoptosis of stressed cells [40,41]. These antioxidants have been shown to repair oxidative damage by activating the endogenous antioxidant defense system. Our results (Figure 3A) show that H_2_O_2_ treatment increased ROS accumulation in cells but that OPH treatment reduced ROS generation significantly in H_2_O_2_-treated cells.

#### 3.2.3. Effect of OPH on Intracellular CAT, SOD and GSH-px Activities

In addition to the accumulation of ROS, treatment with H_2_O_2_ may also cause a considerable reduction in the activity of antioxidant enzymes in PC12 cells. An imbalance between the generation of reactive oxygen species (ROS), free radicals, and the endogenous antioxidant defense systems of the body has also been linked to the etiology of neurodegenerative disorders [42]. The most important antioxidant enzymes found in the brain include CAT, SOD, and GSH-px. Among these antioxidant enzymes, catalase (CAT) and superoxide dismutase (SOD) are the most important in protecting cells from the damaging effects of ROS. GSH-px is essential in protecting membranes from the harmful effects of lipid peroxidation. Therefore, the levels of CAT, SOD, and GSH-px activities in an organism are connected with the capacity of that organism to get rid of free radicals. Based on these studies, we found that several of the associated compounds had undergone modifications [43]. In comparison to the control group, the activities of CAT, GSH-px, and SOD in PC12 cells were reduced to 64.77, 42.94, and 52.84%, respectively, after exposure to H_2_O_2_ (relative value 100%) (Figure 3B–D). However, OPH treatment of PC12 cells resulted in a significant rise in CAT activity (Figure 3B). Additionally, pretreatment with the OPH significantly (*p* < 0.05) enhanced GSH-px activity (Figure 3C), and, compared to the H_2_O_2_-treated group, all the OPH-treated cell groups demonstrated an increase in SOD expression (Figure 3D). The pretreatment with OPH was able to bring about a reversal in these parameter changes, providing evidence that OPH functions as an antioxidant to protect PC12 cells from oxidative damage (Figure 3B–D). Multiple biological compounds with neuroprotective properties are capable of reducing or delaying oxidative-stress-induced apoptosis by up-regulating the endogenous antioxidant defense mechanisms [44]. A previous study revealed that tetrahydroxystilbene glucoside decreases apoptosis by significantly boosting the activities of antioxidant enzymes, including CAT, SOD, and GSH, in PC12 cells [38]. The findings of our study support the hypothesis that increasing the activity of endogenous antioxidant enzymes increases the viability of cells by decreasing the formation of ROS. 

#### 3.2.4. Effect of OPH on Nrf2-Keap1/HO-1 mRNA Expression in H_2_O_2_ Induced PC12 Cells

**Figure 4:**
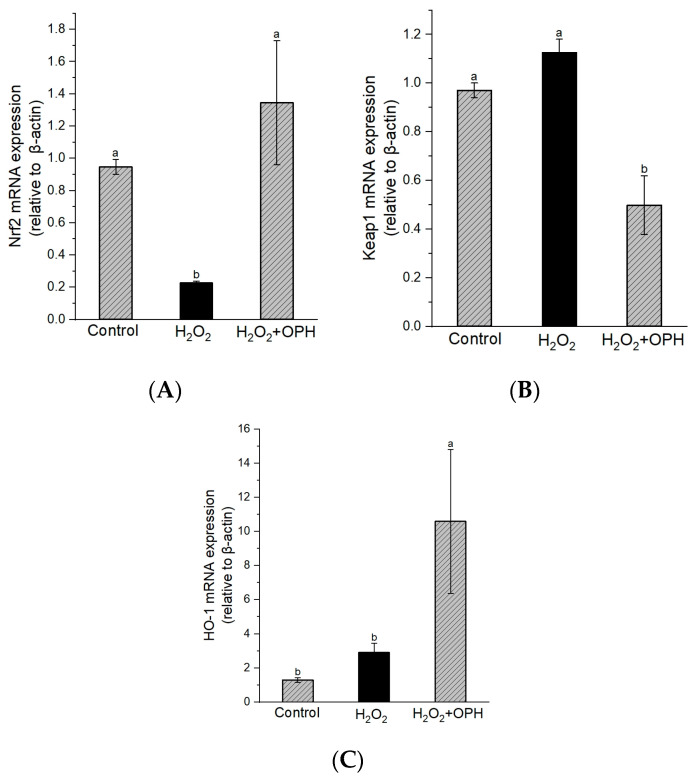
Effect of OPH on Nrf2/Keap1/HO-1 mRNA expression in H_2_O_2_-damaged PC12 cells. (**A**) Nrf2, (**B**) Keap1 and (**C**) HO-1 expression levels, n = 3_._ Different letters (a, b) represent the significant differences among groups (*p* < 0.05).

We explored changes in the Nrf2/Keap1 pathway as a means of further elucidating the mechanism behind the neuroprotective effect of OPH with respect to H_2_O_2_-induced damage to PC12 cells. As indicated in Figure 4A, compared to the control, the Nrf2 level was significantly decreased in H_2_O_2_-damaged cells (*p* < 0.01). However, Nrf2 expression was about 5.9-fold higher in OPH-pretreated (0.5 mg/mL) cells than in H_2_O_2_-damaged cells, although Keap1 expression showed the opposite trend (Figure 4B). On the other hand, as shown in Figure 4C, there were no significant changes in HO-1 mRNA expression between control and H_2_O_2-_damaged cells, but OPH pretreatment showed a significantly increasing trend as the HO-1 expression level increased. In neurodegenerative diseases, Nrf2 transcription factor, which acts through cellular signaling, provides a promising therapeutic target. In particular, the transcription factor Nrf2 is essential for the activation and induction of cytoprotective genes such as antioxidant enzymes and phase-2 genes, which eventually control redox homeostasis and increase cellular antioxidant capacity [45]. Therefore, inhibitors or activators of the Nrf2-Keap1 interaction are recognized as promising therapeutics for neurodegenerative disorders [8]. Antioxidants may increase Nrf2 nuclear translocation in models of neurodegenerative disease, thus activating anti-oxidative enzymes, lowering oxidative stress, and enhancing antioxidative signaling [8,46]. Furthermore, HO-1 has been intensively explored for its powerful neuroprotective and antioxidative capabilities, among several endogenous antioxidant enzymes. A previous study found that walnut-peptide pretreatment stimulated the Nrf2-Keap1/HO-1 pathway, augmented the enhancer activity of antioxidant-responsive elements, upregulated the expression of HO-1, and provided protection against H_2_O_2_-induced neurotoxicity, including apoptosis, oxidative stress, and cell death, in the HT-22 cell line [47]. The findings of this research indicated that OPH significantly increased both the nuclear translocation and the expression of Nrf2 and HO-1 in H_2_O_2_-damaged cells. These findings support a role for OPH in activating the Nrf2/ARE pathway and are thus compatible with the findings that OPH increases the activity of antioxidative enzymes such as CAT, GSH-px, and SOD, as described in the previous section. These findings demonstrate, for the first time, that OPH has a protective effect against H_2_O_2_-induced oxidative stress in PC12 cells through the activation of the Nrf2-mediated pathway.

### 3.3. OPH Peptides Sequence Identification

The peptides that may contribute to OPH’s neuroprotective properties are also quite interesting. The isolation of functional peptides from protein hydrolysates, followed by mass-spectrometry analysis to identify peptide sequences, bioactivities, and structural attributes, constituted a significant aspect of the study [48]. OPH further characterized by LC-MS/MS, with the aim of identifying the putative neuroprotective peptides. According to the results from the Q-executive mass spectrometer, 492 different peptides were detected in OPH (Supplementary Table S2). The fractionation method is not appropriate for screening a significant number of peptides. It is not feasible to analyze the efficacy of all the sub-fractions using an animal model. Therefore, it is necessary to develop an effective strategy for screening large numbers of bioactive peptides. The application of the peptidomics approach in protein hydrolysate analysis has the potential to significantly decrease the number of separation stages needed and thus to increase the rate of peptide screening. The combination of molecular docking and PeptideRanker was extensively employed to identify potentially active peptides in this study. Evaluation of PeptideRanker scores, relative peak area, and matching entries in the protein database facilitated the screening of neuroprotective peptides anticipated to have bioactivity. Finally, 25 peptides were selected based on their high PeptideRanker scores (>0.8) and relative peak areas (>10^7^). The peptide sequence for the chosen peptides, as well their peptide’s parent proteins, molecular masses, relative peaks, and PeptideRanker scores, are presented in Table 1. These peptides range in length from 8 to 15, with the majority falling within the octal-to-decapeptide range.

### 3.4. Interaction of Peptides with Keap1 Protein in a Molecular-Docking Simulation

After considering multiple factors such as the protein database, the PeptideRanker score, and the relative peak area, we selected 25 of the peptides derived from OPH for subsequent Keap1-docking analysis to assess their potential as neuroprotective peptides. The Keap1 Vina scores of the chosen peptides range from −2.70 to −10.10 kcal/mol (Table 1). The peptides DFVADHPFLF, HGQNFPIL, and RDFPITWPW were selected for further docking testing due to their low Vina scores (−10.1, −10.1, and −10, respectively) and interactions (Table 2). These peptides exhibit the potential to modulate the Keap1–Nrf2 signaling pathway, a critical regulator of cellular antioxidant defenses. The Nrf2 pathway’s interaction with Keap1 results in alterations that influence Keap1–Nrf2 dynamics, subsequently enhancing stability and triggering the expression of target genes. Previous reports have indicated that peptides (SGFDAE, DKK, and DWW) can enhance the functions of antioxidant-defense enzymes by interacting with Keap1 through hydrogen bonding during molecular docking [49]. Therefore, we postulated that oat-derived peptides could potentially activate Nrf2 and disrupt the Keap1–Nrf2 interaction, and this hypothesis was subsequently validated through molecular docking. The peptides DFVADHPFLF, HGQNFPIL, and RDFPITWPW were identified as potential inhibitors of Keap–Nrf2 based on their Vina affinity scores. Subsequent docking analysis demonstrated the presence of strong conventional and carbon-hydrogen bonds DFVADHPFLF, HGQNFPIL, RDFPITWPW, and Keap1 (Supplementary Figure S1A–C). Additionally, π–alkyl and π–π interactions were also found between HGQNFPIL, RDFPITWPW, and Keap1. It is interesting to note that peptides containing Trp (W) and Phe (F) exhibited a strong affinity for Keap1. These peptides may interact with the target protein due to the presence of the benzene ring, which induces hydrophobic interactions, strong hydrogen bonding, and π–π stacking. Our research results align with those of a previous study, which also concluded that peptides containing aromatic amino acids in their sequences have a neuroprotective effect [50]. Furthermore, the presence of Pro (P), Leu (L), Ile (I), and Ala (A) in the sequence enhances the hydrophobicity of the peptide, potentially promoting its interactions with Keap1’s active site. The high affinities of all three peptides for Keap1 may be attributed to the presence of charged amino acids (Asp D, His H, and Arg R) at the N-terminal. These amino acids form hydrogen and carbon bonds with the active site of Keap1. In light of these observations, it can be concluded that the presence of these residues and hydrogen bonding are significant factors in the inhibition of Keap1 by the peptides DFVADHPFLF, HGQNFPIL, and RDFPITWPW.

### 3.5. Effects of HL-8, RW-9, and DF-10 Peptides on Zebrafish Behavior in T-Maze Testing

**Figure 5:**
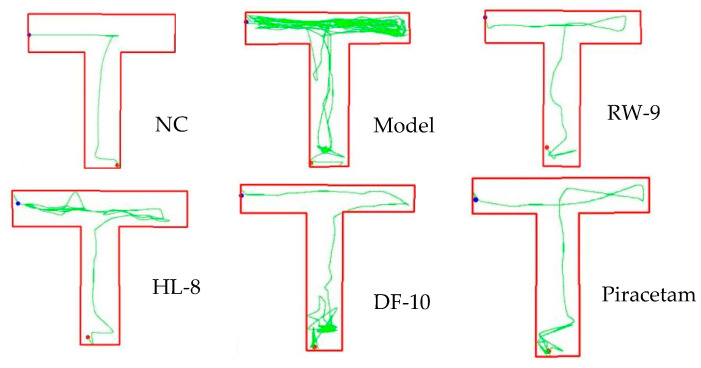
Behavior of zebrafish in T-maze testing.

The zebrafish (*Danio rerio*) is a cost-effective and valuable model organism used for screening drugs for neurodegenerative diseases. Its usefulness is the result of its low maintenance requirements, high reproductive capacity, and genetic similarity to humans; the zebrafish genome has over 87% homology with the human genome. This last characteristic makes it a relevant model for investigating behavioral and functional parameters related to pathogenesis in humans. Zebrafish are extensively employed to explore molecular mechanisms of neurodevelopment and cognitive processes such as learning and memory. Notably, studies have demonstrated that cotinine, 6-hydroxy-L-nicotine, and curcumin can improve cognitive function following scopolamine-induced impairment in zebrafish [51,52]. Therefore, the selected neuroprotective peptides HL-8, RW-9, and DF-10, having been chosen based on in silico calculations, were subsequently tested in a zebrafish model with scopolamine-induced memory impairment. The T-maze test revealed that scopolamine led to deficits in memory and spatial recognition, as seen in prolonged escape latency and increased travel distance (Figure 5). These results aligned with earlier research indicating the memory-disrupting effects of scopolamine [51]. Notably, HL-8, RW-9, DF-10, and piracetam mitigated these effects, reducing distance and escape latency compared to scopolamine exposure alone (*p* < 0.05). Additionally, scopolamine reduced target-reservoir time, while the peptide treatments increased it significantly (*p* < 0.05), suggesting memory restoration (Table 3). Thus, HL-8, RW-9, and DF-10 peptides effectively alleviated scopolamine-induced memory dysfunction, as shown in the zebrafish T-maze test.

### 3.6. Effects of HL-8, RW-9, and DF-10 Peptides on Acetylcholinesterase Activity in the Zebrafish Brain

**Figure 6:**
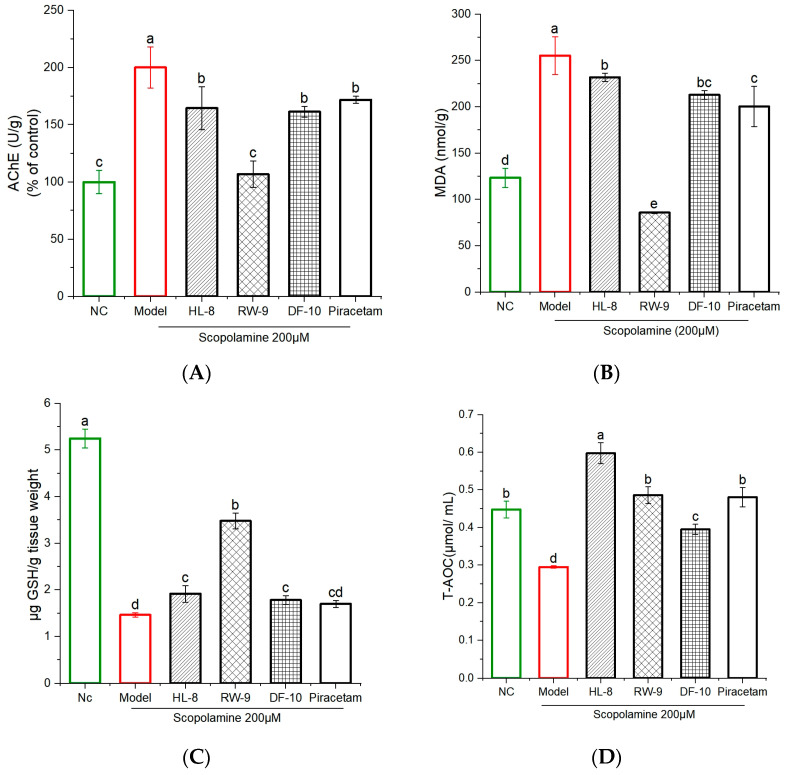
Effects of HL-8, RW-9, and DF-10 peptides on levels of acetylcholinesterase and oxidative stress in the brains of scopolamine-exposed zebrafish. (**A**) AChE, (**B**) MDA, (**C**) GSH, (**D**) T-AOC; n = 3. The data are presented as the mean ± S.D., and different letters (a, b, c, d, e) indicate significant differences among groups (*p* < 0.05).

The cholinergic system is essential for regulating memory and learning. During brain aging, cholinergic neurons undergo degeneration, leading to reduced nerve impulses and impaired signal transmission. This dysfunction contributes to the development of learning impairments and memory disorders [53,54]. Acetylcholine (ACh) is an essential neurotransmitter that plays a fundamental role in cognitive processes and the regulation of memory. It is widely present in the central nervous system (CNS) and is degraded into choline and acetate by the enzyme AChE [55]. Excessive degradation of ACh prevents it from effectively binding to its receptors on the postsynaptic membrane. This interference disrupts the transmission of nerve signals between synapses, leading to memory loss [56]. Therefore, one potential therapeutic approach involves increasing the levels of ACh by using AChE inhibitors, which prevent the breakdown of ACh in the brain [57]. Our research demonstrated that zebrafish exposed to scopolamine exhibited a significant increase (*p* < 0.05) in levels of AChE compared to the control group. Interestingly, the administration of three peptides (HL-8, RW-9, and DF-10) and piracetam effectively mitigated the effect of scopolamine, leading to a significant reduction in AChE activity in the brains of scopolamine-exposed zebrafish (Figure 6A). This result suggests that oat peptides have the potential to alleviate scopolamine-induced memory impairment in zebrafish by modulating the AchE level. Notably, the maximum reduction in AChE activity was observed in the group administered RW-9 peptide; however, there were no significant differences among the HL-8-, DF-10-, and piracetam-treated groups. Previous findings have indicated that walnut peptides (SGFDAE and FY) enhance cholinergic activity, improving memory in scopolamine-exposed mice [50]. Similarly, *Rosmarinus officinalis* essential oil improved memory in scopolamine-exposed zebrafish by regulating AChE activity [58].

### 3.7. Effects of HL-8, RW-9, and DF-10 Peptides on the Levels of MDA, GSH, and T-AOC in the Zebrafish Brain

In the studies of oxidative-stress parameters, scopolamine exposure significantly reduced the levels of GSH and T-AOC in the zebrafish brain as compared to the unexposed control. This reduction indicates a detrimental impact on the antioxidant defense system. Administration of HL-8, RW-9, DF-10, and piracetam mitigated oxidative stress by increasing the levels of GSH and T-AOC in the zebrafish brain (*p* < 0.05). Additionally, the marked increase in MDA levels observed in the treated group suggests that scopolamine triggered lipid peroxidation in the brain, while supplementation with HL-8, RW-9, DF-10, and piracetam significantly reversed this effect (*p* < 0.05), with a reduction in lipid peroxidation (Figure 6B–D). The results reveal that treatment with RW-9 was superior compared to HL-8 and DF-10 at mitigating the decline in antioxidant capacity and increase in MDA levels in the zebrafish brain that were induced by scopolamine exposure. As a metabolically active organ with heightened oxygen consumption, the brain possesses limited antioxidant defenses and is susceptible to oxidative damage from free radicals [59]. Oxidative stress within the brain triggers neurodegeneration, a hallmark of Alzheimer’s disease [60]. Individuals with Alzheimer’s disease exhibit significant deficits in their antioxidant system, rendering it incapable of effectively neutralizing elevated levels of oxidative-stress indicators [61]. Furthermore, the uncontrolled oxidative stress evident during the progression of neurodegeneration disrupts the homeostasis of the cholinergic system. Our study revealed a noticeable reduction in GSH and T-AOC levels, alongside a significant elevation in MDA levels, in the brains of zebrafish exposed to scopolamine. These changes collectively indicate the onset of oxidative stress in the brain. However, the administration of peptides effectively reversed these changes. Moreover, a close interrelation exists between oxidative stress and inflammation, with oxidative stress partially promoting the progression of inflammation [62]. In a previous study, walnut-protein hydrolysates were shown to have neuroprotective effects in scopolamine-exposed mice by reducing oxidative stress in the brain [50]. Similarly, another study showed memory improvement in lipopolysaccharide-exposed mice after administration of walnut LPF, GVYY, and APTLW peptides, which inhibited oxidative stress and inflammatory responses in the brain [62]. Furthermore, RPVKRKKGWPKGVKRGPPKW (RW-20) from histone acetyltransferases has been shown to have neuroprotection effects through a reduction in oxidative stress and improved antioxidant-enzyme activity in zebrafish larvae [63]. Interestingly, this peptide has a structure similar to that of our peptide RW-9, as both peptides have the same amino acids on their NH_2_ and COOH ends, and RW-9 showed neuroprotective activity in the current study. Notably, treatment with RW-9 resulted in reduced MDA levels and increased GSH levels in zebrafish brains compared to treatment with DF-10 and HL-8. The greater effect of RW-9 can be partially attributed to its robust ability to scavenge radicals. Previous research has shown that the amino acids Trp (W) and Tyr (Y) can inhibit or delay free-radical chain reactions by providing hydrogen atoms to free radicals [64]. These findings underscore the potential of oxidative-stress modulation as a potent mechanism through which oat peptides can offer neuroprotective effects.

### 3.8. Effects of HL-8, RW-9, and DF-10 Peptides on the Levels of Proinflammatory Cytokines in the Zebrafish Brain

**Figure 7:**
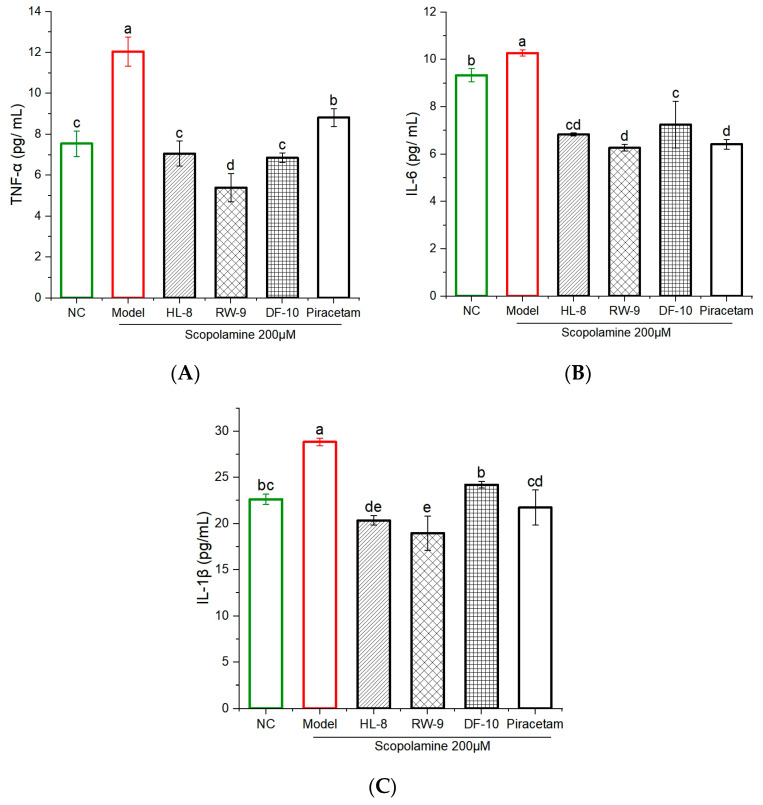
Effects of HL-8, RW-9, and DF-10 peptides on the inflammatory cytokines in the brains of Scopolamine-induced Zebrafish. (**A**) TNF-α, (**B**) IL-6, (**C**) IL-1β, n = 4. The data are presented mean ± S.D and different letters “a, b, c, d, e” among groups represents a significant difference (*p* < 0.05).

Proinflammatory cytokines have been found to induce acute and chronic inflammation, leading to the development of neurodegenerative disorders [65]. This study investigated the impacts of HL-8, RW-9, and DF-10 on the production of TNF-α, IL-1β, and IL-6 in scopolamine-exposed zebrafish. The results shown in Figure 7A–C reveal an increase in the production of TNF-α, IL-1β, and IL-6 in zebrafish brains after scopolamine exposure. However, administration of HL-8, RW-9, and DF-10 led to a significant reduction in TNF-α production, with levels decreasing from 12.04 ± 0.71 to 7.05 ± 0.62, 5.39 ± 0.7, and 6.85 ± 0.23 pg/mL, respectively (Figure 7A). Likewise, the levels of IL-6 were significantly lower in the groups that received peptides and piracetam compared to the group served as a control (Figure 7B). In addition, treatment with all three peptides resulted in a noticeable reduction in the level of IL-1β, whereas the scopolamine insult led to a significant increase in IL-1β levels (28.84 ± 0.41 pg/mL) (Figure 7C). The anti-inflammatory properties of HL-8, RW-9, and DF-10 may be attributed to their hydrophobic nature and high content of the amino acids Val (V), Phe (F), Ala (A), Pro (P), Leu (L), and Trp (W) in their peptide sequences. It is widely acknowledged that hydrophobic amino acids are important for anti-inflammatory activity. In fact, a significant number of peptides known for their anti-inflammatory properties have been found to possess a substantial proportion of hydrophobic amino acids [66]. The anti-inflammatory effects of peptides containing hydrophobic amino acids have been demonstrated in various studies. Relevant peptide include QCQCAVQGGL from *Crassostrea gigas*, QCQQAVQSAV from *Ruditapes philippinarum*, GVSLLQQFFL from *Mytilus coruscus*, and GVYY and APTLW from walnut protein [62,67,68,69]. Additionally, the HL-8, RW-9, and DF-10 peptides were found to have aromatic and hydrophobic amino acids in their sequence. Previous studies have shown that aromatic amino acids (F and W) in peptides can also influence their anti-inflammatory capacity [70]).

### 3.9. Effects of HL-8, RW-9, and DF-10 Peptides on mRNA Expression of Nrf2, Bdnf and Erg-1 in Zebrafish Brain

**Figure 8:**
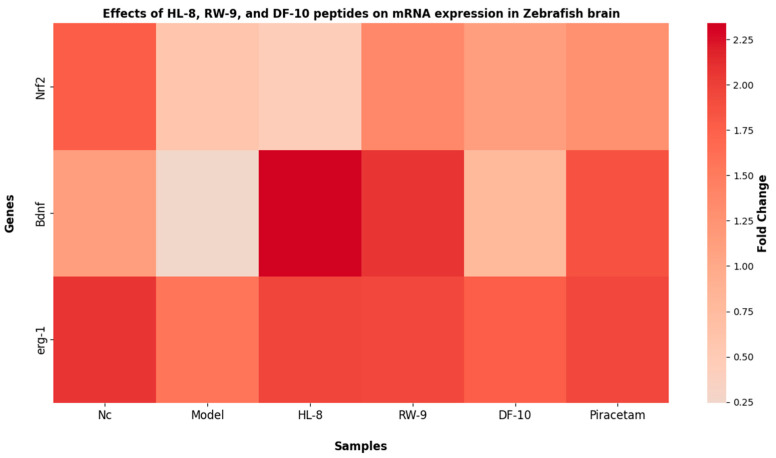
Effects of HL-8, RW-9, and DF-10 peptides on the Nrf2, Bdnf, and Erg1 gene expression zebrafish brains.

In order to further elucidate the cognitive and memory-improving effects of oat peptides, the mRNA expression of Nrf2, Bdnf, and Erg1 was studied (Figure 8). The administration of scopolamine resulted in a significant reduction (*p* < 0.05) in the mRNA expression of Nrf2, Bdnf, and Erg1 compared to the control group. Conversely, a significant upregulation in the mRNA expression of these specific genes was observed subsequent to the administration of HL-8, RW-9, and DF-10 peptides. The Nrf2–Keap1 pathway, a vital defense against oxidative stress in neurodegenerative disorders, involves Nrf2 translocating into the nucleus upon dissociation from Keap1 and binding to antioxidant response elements (ARE), subsequently regulating the expression of antioxidant enzymes [71,72]. Moreover, the Nrf2 pathway has been associated with countering oxidative stress-induced cognitive impairment both in vitro and in vivo [46]. In the current study, an increase in the level of Nrf2 mRNA was observed in the zebrafish brain after peptide treatment, suggesting that oat peptides’ neuroprotective effects might be linked to activation of the Nrf2 pathway. Additionally, extensive evidence supports the involvement of Bdnf in the processes of memory formation and consolidation. Research has found that Bdnf exerts its influence on the proliferation and differentiation of neural cells in the central nervous system by interacting with the pCREB signaling pathway [73], facilitating cognitive function through CREB activation and subsequent Bdnf expression. Conversely, impaired activation contributes to long-term memory issues associated with neurodegeneration [74]. The results of our study indicate that the studied peptides upregulate Bdnf mRNA expression. Additionally, HL-8, RW-9, and DF-10 were found to mitigate oxidative stress and cholinergic dysfunction in the zebrafish brain. These effects indicate that oat peptides have the potential to preserve cognitive function by modulating the Bdnf pathway in a zebrafish model of scopolamine-induced impairment. Early growth response protein 1 (Erg1) functions as a crucial transcription factor involved in various neuronal activities, ranging from neurotransmission and synaptic plasticity to complex cognitive processes like memory and learning. The expression of Erg1 in neurons is triggered by activity-dependent synaptic plasticity that occurs during the learning process [75]. The present study assessed the expression of the Erg1 gene, and the results indicate a significant reduction in erg1 mRNA levels in the brains of zebrafish exposed to scopolamine relative to the control group. However, administration of HL-8, RW-9, and DF-10 peptides enhances Erg1 mRNA expression in the brains of zebrafish subjected to scopolamine treatment. Scopolamine administration has been observed to impair memory consolidation while also prompting the downregulation of various immediate early genes, including erg1 [76]. These findings indicate that oat peptides may have the potential to improve cognitive abilities, including memory and learning, through upregulation of Nrf2, Bdnf, and erg1 gene expression in zebrafish brains.

### 3.10. Correlation Analysis of Behavioral, Genetic and Biochemical Parameters

**Figure 9:**
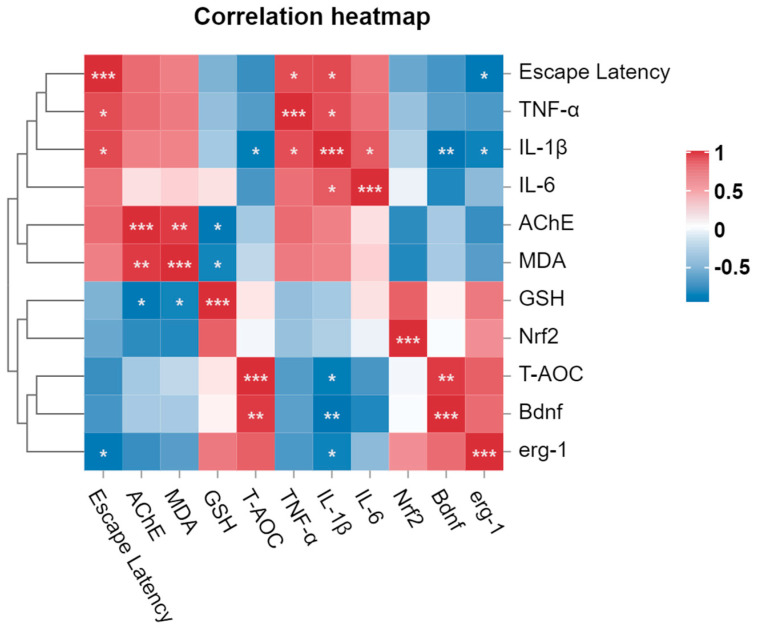
Correlation analysis of behavioral, genetic, and biochemical parameters. In the graph (*, **, and ***) represents the moderate, strong, and very strong correlation between different parameters, respectively.

The correlation analysis elucidated the associations among various parameters in the study (Figure 9). Specifically, memory improvement, as evidenced by reduced escape latency in the T-maze, exhibited a negative correlation with gene expressions of Bdnf, Nrf2, and Erg1. This result suggests that upregulation of the expression of these genes could potentially lead to improved memory functions. Similarly, escape latency was strongly positively correlated with levels of AChE, MDA, and inflammatory cytokines, indicating that poor memory function was associated with an increase in these parameters. Among biochemical parameters, a strong positive correlation (r = 0.9479) between AChE and MDA was observed, indicating increased activity of the AChE enzyme in the zebrafish brain when MDA levels are elevated. Moreover, the results of this study included highly negative correlations between the MDA level and levels of the components of the antioxidant defense system, including GSH (r = −0.8232) and T-AOC (r = −0.2525). These outcomes suggest that the improvement in the functioning of the antioxidant defense system associated with the HL-8, RW-9, and DF-10 peptides is strongly correlated with a reduction in the MDA level. Furthermore, we observed a positive correlation between levels of antioxidant components, including GSH and T-AOC, and Nrf2 gene expression, while MDA was found to be strongly negatively (r = −0.7990) correlated with Nrf2 gene expression. Meanwhile, the inflammatory cytokines, including TNF-α, IL-1β, and IL-6, had a strong negative correlation (r = −0.6499, 0.9263, and 0.8090, respectively) with Bdnf gene expression. It has been previously reported that Bdnf might affect the inflammatory response by downregulating proinflammatory cytokines and cyclooxygenase-2 in microglial cells [77]. According to our correlation analysis, all three peptides and piracetam exhibited memory-improving and neuroprotective activities as compared to the scopolamine-exposed control group (Supplementary Figure S2). However, the results of RW-9 treatment were observed to have a strong correlation with the results from the NC group, while the results of DF-10 treatment significantly correlated with results from the piracetam group.

## 4. Conclusions

This study found that oat protein hydrolysates (OPH) exhibit antioxidant properties in vitro and exert neuroprotective effects in PC12 cells exposed to hydrogen peroxide-induced oxidative stress. After peptidome analysis, 25 promising peptides were selected based on their abundance and peptide-ranker scores. Among these peptides, DF-10, HL-8, and RW-9, due to their high keap1-inhibitory activity in the molecular-docking analysis, were selected for further assessment of neuroprotective capacity using the zebrafish model. These peptides were found to improve behavioral performance in scopolamine-exposed zebrafish, reduce AChE activity, mitigate oxidative stress, and decrease levels of inflammatory cytokines, including TNF-α, IL-6, and IL-1β, in the zebrafish brain. The peptides’ anti-oxidative and anti-inflammatory effects are attributed to the presence of hydrophobic, aromatic amino acids Phe (F), Val (V), Ala (A), Pro (P), and Leu (L), along with arginine (R) and tryptophan (W), all of which are associated with neuroprotection. This study highlights that the neuroprotective action of peptides involves upregulation of neurotropic-related (Bdnf), antioxidant-related (Nrf2), and neural-activity-related (Erg1) genes. Further research on these neuroprotective peptides should be carried out to explore gut-flora modulation and other related biomarkers to enable deeper understanding of neuroprotection through the gut-brain axis.

## Figures and Tables

**Table 1:** Identification of Peptides with Potential Neuroprotective Properties Isolated from OPH.

Peptide Sequence	Molecular Mass (Da)	NCBI Accession Number *^(a)^*	Peptide Ranker Score *^(b)^*	Peak Intensity	Binding Affinity (kcal/mol)
ADHPFLFL	958.49	AAD31175	0.968736	3.9672 × 10^7^	+
APSKDAPMF	962.45	ANQ69423	0.851488	4.553 × 10^8^	−8
APSKDAPMFVM	1192.56	QBA82502	0.8609	1.5592 × 10^9^	−5.1
DFGWGRPVFM	1210.55	QDC27805	0.954285	4.4557 × 10^7^	+
DFVADHPFLF (DF-10)	1206.57	AAD31175	0.923659	1.5872 × 10^9^	−10.1
DFVADHPFLFL	1319.65	AAD31175	0.934308	6.1868 × 10^8^	+
DHHDRFMPF	1200.51	AEL03787	0.90309	4.4413 × 10^8^	+
FIDNIFRF	1070.55	ABH02581	0.956105	5.3257 × 10^7^	−8.9
FNPDKSPAYPIRF	1550.78	AAF80276	0.883611	1.7912 × 10^9^	+
FSLAPLVPRL	1111.67	AAA86837	0.875095	5.1651 × 10^7^	−8.2
GDGIIYPWETFRGL	1622.80	G1JSL4.1	0.883918	4.4366 × 10^7^	---
GWVANKGEWILL	1384.75	G1JSL4.1	0.845169	1.2307 × 10^8^	+
HGQNFPIL (HL-8)	924.48	CAA54152	0.811976	1.4818 × 10^9^	−10.1
IDFVADHPFLFL	1432.73	AAD31175	0.908569	4.399 × 10^8^	---
KDFPLTWPW	1188.59	AFJ91164	0.95263	1.3177 × 10^8^	−8.5
PGGGVRLDPGKSWAL	1508.81	AAB02259	0.830548	1.2107 × 10^8^	---
QGLQFLKPF	1076.60	P12615	0.875919	9.3927 × 10^8^	−8.6
RDFPITWPW (RW-9)	1216.60	P27919	0.921925	3.7349 × 10^9^	−10.0
SGVFTPKF	881.46	CAA54152	0.841774	1.657910^9^	−8.5
SIQHELGGFF	1133.55	UKZ80143	0.814202	8.4774 × 10^8^	−2.7
VADHPFLFL	1057.55	AAD31175	0.929798	3.7088 × 10^8^	−9.6
VWPGALPGGGVR	1164.64	AAB02259	0.83325	3.4743 × 10^7^	−9.8
LIPFPRLH	991.59	QBA82506	0.809332	1.4957 × 10^8^	−8.7
NLIPFPRL	968.58	QBA82506	0.893781	3.4329 × 10^7^	−7.7
NLIPFPRLH	1105.63	QBA82506	0.796125	3.6755 × 10^8^	−7.7

*^(a)^* NCBI (https://www.ncbi.nlm.nih.gov/, accessed on 15 August 2022). *^(b)^* PeptideRanker (http://distilldeep.ucd.ie/PeptideRanker/, accessed on 15 August 2022). (binding affinity (+) and (---) represent poor docking with positive binding affinity and no confirmation of docking, respectively).

**Table 2:** Details of the interaction of Keap1 residues with peptides.

Parameters	Keap1–DFVADHPFLF	Keap1–HGQNFPIL	Keap1–RDFPITWPW
hydrogen bond	SER555, LEU557, VAL463, VAL418, VAL418, VAL465, VAL512	VAL465-GLN3, VAL512, VAL608, THR560, VAL606, ASN4-PHE5, GLY367, ILE559, ILE559,	SER602, ARG415, ASN414, SER363, ASN382, ILE559, ILE559, VAL369, VAL420
carbon hydrogen bond	ARG415, ARG415, GLY509, GLY509, Gly462, GLY511, GLY464, GLY417, GLY417, GLY367	SER508, ARG415, GLY364, GLY464, Ala366, GLY558, ALA607	ARG415, SER365, GLY462, GLY509, AL510, GLY558, VAL606, VAL418, CYS368, VAL369
π–alkyl		CYS368, VAL420	ALA556, CYS513, VAL514, VAL467
alkyl		CYS513, VAL514	ALA556, VAL512, ALA607
π–π stacked			TYR525
π–donor		SER508	VAL514, VAL467

**Table 3:** Behavior of zebrafish in T-maze testing.

Treatment Group	Escape Latency (S)	Total Distance (mm)	Target Time (S)
NC	6.63 ± 2.06 ^d^	1035.89 ± 117.86 ^d^	173.36 ± 2.1 ^a^
Model	85.38 ± 18.52 ^a^	11,283.03 ± 2138.72 ^a^	94.61 ± 18.5 ^d^
HL-8	16.72 ± 2.11 ^bc^	1728.3987 ± 205.5 ^cd^	163.27 ± 2.11 ^bc^
RW-9	9.08 ± 2.40 ^cd^	1249.01 ± 213.9 ^d^	170.91 ± 2.4 ^ab^
DF-10	21.09 ± 5.58 ^b^	3319.97 ± 103.8 ^b^	158.90 ± 5.6 ^c^
Piracetam	16.53 ± 4.07 ^bc^	2374.7217 ± 549.6 ^bc^	163.46 ± 4.1 ^bc^

Different letters (a, b, c, d) represent the significant differences among groups (*p* < 0.05).

## Data Availability

All relevant data are within the paper and attached supplementary file.

## Supplementary Materials

The following supporting information can be downloaded at: https://www.mdpi.com/article/10.3390/nu16010117/s1, Figure S1: Molecular docking interaction of peptides; Figure S2: Correlation analysis of samples and biochemical parameters; Table S1: Primers sequences for RT-qPCR analysis; Table S2: Peptide’s profile of OPH identified by LC-MS/MS.

## References

[1] Huang Q., Liao C., Ge F., Ao J., Liu T. (2022). Acetylcholine bidirectionally regulates learning and memory. J. Neurorestoratol..

[2] Singh A., Kukreti R., Saso L. (2019). Oxidative Stress: A Key Modulator in Neurodegenerative Diseases. Molecules.

[3] Kim J.M., Park C.H., Park S.K., Seung T.W., Kang J.Y., Ha J.S., Lee D.S., Lee U., Ki D.O., Heo H.J. (2017). Ginsenoside Re Ameliorates Brain Insulin Resistance and Cognitive Dysfunction in High Fat Diet-Induced C57BL/6 Mice. J. Agric. Food Chem..

[4] Foret M.K., Carmo S.D., Lincon R., Greene L.E., Zhang W., Cuello A.C., Cosa G. (2019). Effect of antioxidant supplements on lipid peroxidation levels in primary cortical neuron cultures. Free Radic. Biol. Med..

[5] Giudetti A.M., Salzet M., Cassano T. (2018). Editorial Oxidative Stress in Aging Brain: Nutritional and Pharmacological Interventions for Neurodegenerative Disorders. Hindawi Oxidative Med. Cell. Longev..

[6] Yuan J., Amin P., Ofengeim D. (2019). Necroptosis and RIPK1-mediated neuroinflammation in CNS diseases. Nat. Rev. Neurosci..

[7] Simpson D.S.A. (2020). ROS Generation in Microglia: Understanding Oxidative Stress and Inflammation in Neurodegenerative Disease. Antioxidants.

[8] Lee J., Song K., Huh E., Sook M., Shik Y. (2018). Redox Biology Neuroprotection against 6-OHDA toxicity in PC12 cells and mice through the Nrf2 pathway by a sesquiterpenoid from Tussilago farfara. Redox Biol..

[9] Buendia I., Michalska P., Navarro E., Gameiro I., Egea J., León R. (2016). Pharmacology & Therapeutics Nrf2–ARE pathway: An emerging target against oxidative stress and neuroin fl ammation in neurodegenerative diseases. Pharmacol. Ther..

[10] Widowati M.W., Prahastuti S., Hidayat M., Hasiana S.T., Wahyudianingsih R., Afifah E., Kusuma H.S.W., Rizal R., Subangkit M. (2022). Protective Effect of Ethanolic Extract of Jati Belanda (*Guazuma ulmifolia* L.) by Inhibiting Oxidative Stress and Inflammatory Processes in Cisplatin-induced Nephrotoxicity in Rats. Pak. Vet. J..

[11] Bae I.K., Kim K.J., Choi J.S., Choi Y.I., Ha J.H. (2019). Quality properties and storage characteristics of pyeonyuk with different additional levels of turmeric powder. Food Sci. Anim. Resour..

[12] Pokorny J.P. (2007). Are natural antioxidants better-and safer-than synthetic antioxidants?. Eur. J. Lipid Sci. Technol..

[13] Hussein M.M.A., Gaafar S.F. (2022). Histidine-Dipeptides in Relation to Diabetes and Obesity. Int. J. Vet. Sci..

[14] Kiliç A., Gökhan K.D., Sözmen A., Uysal E.Y. (2022). Liver Histology and Biochemistry of Exposed Newborn and Infant Rats with Experimental Aflatoxicosis. Pak. Vet. J..

[15] Cai M., Dou B., Pugh J.E., Lett A.M., Frost G.S. (2021). The impact of starchy food structure on postprandial glycemic response and appetite: A systematic review with meta-analysis of randomized crossover trials. Am. J. Clin. Nutr..

[16] Chen H., Zhao M., Lin L., Wang J., Waterhouse D.S., Dong Y., Zhuang M., Su G. (2015). Identification of antioxidative peptides from defatted walnut meal hydrolysate with potential for improving learning and memory. Food Res. Int..

[17] Kim E.K., Lee S.J., Hwang J.W., Kin C.G., Choi D.K., Lim B.O., Kanh H., Moon S.O., Jeon B.T., Park P.J. (2011). In vitro investigation on antioxidative effect of Inonotus obliquus extracts against oxidative stress on PC12 cells. J. Appl. Biol. Chem..

[18] Sudhakar S., Nazeer R.A. (2015). Structural characterization of an Indian squid antioxidant peptide and its protective effect against cellular reactive oxygen species. J. Funct. Foods.

[19] Asala C.A.T.M., Rowaiye A.B., Salami S.A., Baba-onoja M., Abatan M.O., Ocheja B.O., Ada A.G., Ogu A.M. (2022). The Antioxidant and Hematopoietic Effects of the Methanolic Extract Fractions of *Ocimum basilicum* in Acetaminophen-Induced Albino Rats. Int. J. Vet. Sci..

[20] Majhi S. (2021). Applications of ultrasound in total synthesis of bioactive natural products: A promising green tool. Ultrason. Sonochem..

[21] Li W., Chen W., Ma H., Wu D., Zhang Z., Yang Y. (2022). Ultrasonics Sonochemistry Structural characterization and angiotensin-converting enzyme (ACE) inhibitory mechanism of Stropharia rugosoannulata mushroom peptides prepared by ultrasound. Ultrason. Sonochem..

[22] Rafique H., Dong R., Wang X., Alim A., Aadil R.M., Li L., Zou L., Hu X. (2022). Dietary-Nutraceutical Properties of Oat Protein and Peptides. Front. Nutr..

[23] Yue J., Gu Z., Zhu Z., Yi J., Ohm J.B., Chen B., Rao J. (2021). Impact of defatting treatment and oat varieties on structural, functional properties, and aromatic profile of oat protein. Food Hydrocoll..

[24] You L., Zhao M., Regenstein J.M., Ren J. (2011). In vitro antioxidant activity and in vivo anti-fatigue effect of loach (*Misgurnus anguillicaudatus*) peptides prepared by papain digestion. Food Chem..

[25] Esfandi R., Willmore W.G., Tsopmo A. (2019). Peptidomic analysis of hydrolyzed oat bran proteins, and their in vitro antioxidant and metal chelating properties. Food Chem..

[26] Ma S., Liu H., Jiao H., Wang L., Chen L., Liang J., Zhao M., Zhang X. (2012). Neuroprotective effect of ginkgolide K on glutamate-induced cytotoxicity in PC12 cells via inhibition of ROS generation and Ca^2+^ influx. Neurotoxicology.

[27] Hroudová J., Singh N., Fišar Z. (2014). Mitochondrial dysfunctions in neurodegenerative diseases: Relevance to alzheimer’s disease. Biomed Res. Int..

[28] Vlieghe P., Lisowski V., Martinez J., Khrestchatisky M. (2010). Synthetic therapeutic peptides: Science and market. Drug Discov. Today.

[29] Wang S., Su G., Zhang Q., Zhao T., Liu Y., Zheng L., Zhao M. (2018). Walnut (*Juglans regia*) Peptides Reverse Sleep Deprivation-Induced Memory Impairment in Rat via Alleviating Oxidative Stress. J. Agric. Food Chem..

[30] Zhao T., Su G., Wang S., Zhang Q., Zhang J., Zheng L., Sun B., Zhao M. (2017). Neuroprotective Effects of Acetylcholinesterase Inhibitory Peptides from Anchovy (*Coilia mystus*) against Glutamate-Induced Toxicity in PC12 Cells. J. Agric. Food Chem..

[31] Wu Q., Zhang X., Jia J., Kuang C., Yang H. (2018). Effect of ultrasonic pretreatment on whey protein hydrolysis by alcalase: Thermodynamic parameters, physicochemical properties and bioactivities. Process Biochem..

[32] Jia J., Ma H., Zhao W., Wang Z., Tian W., Luo L., He R. (2010). The use of ultrasound for enzymatic preparation of ACE-inhibitory peptides from wheat germ protein. Food Chem..

[33] Wang B., Ma H., Jia J., He R., Luo L., Pan Z. (2015). Ultrasonic Treatment Effect on Enzymolysis Kinetics and Activities of ACE-Inhibitory Peptides from Oat-Isolated Protein. Food Biophys..

[34] He L., Gao Y., Wang X., Han L., Yu Q., Shi H., Song R. (2021). Ultrasonics Sonochemistry Ultrasonication promotes extraction of antioxidant peptides from oxhide gelatin by modifying collagen molecule structure. Ultrason. Sonochem..

[35] Resendiz-Vazquez J.A., Ullao J.A., Urias-Silvas J.E., Bautista-Rosales P.U., Ramirez-Ramirez J.C., Rosas-Ullao P., Gonzalez-Torres L. (2017). Effect of high-intensity ultrasound on the technofunctional properties and structure of jackfruit (*Artocarpus heterophyllus*) seed protein isolate. Ultrason. Sonochem..

[36] Yang X., Li Y., Li S., Oladejo A.O., Ruan S., Wang Y., Huang S., Ma H. (2017). Effects of ultrasound pretreatment with different frequencies and working modes on the enzymolysis and the structure characterization of rice protein. Ultrason. Sonochem..

[37] Daskaya-Dikmen C., Yucetepe A., Karbancioglu-Guler F., Daskaya H., Ozcelik B. (2017). Angiotensin-I-converting enzyme (ACE)-inhibitory peptides from plants. Nutrients.

[38] Zhang L., Huang L., Li X., Liu C., Sun X., Wu L., Li T., Yang H., Chen J. (2017). Potential molecular mechanisms mediating the protective effects of tetrahydroxystilbene glucoside on MPP+-induced PC12 cell apoptosis. Mol. Cell. Biochem..

[39] Ott M., Gogvadze V., Orrenius S. (2007). Mitochondria, oxidative stress and cell death. Apoptosis.

[40] Tang X., Ren Y., Zhou C., Yang C., Gu H. (2012). Neurochemistry International Hydrogen sulfide prevents formaldehyde-induced neurotoxicity to PC12 cells by attenuation of mitochondrial dysfunction and pro-apoptotic potential. Neurochem. Int..

[41] Liu J., Chen Z., He J., Zhang Y., Zhang T., Jiang Y. (2014). Function Anti-oxidative and anti-apoptosis effects of egg. Food Funct..

[42] Santos C.Y., Snyder P.J., Wu W.C., Zhang M., Echeverria A., Alber J. (2017). Pathophysiologic relationship between Alzheimer’s disease, cerebrovascular disease, and cardiovascular risk: A review and synthesis. Alzheimer’s Dement. Diagn. Assess. Dis. Monit..

[43] Chu Q., Chen M., Song D., Li X., Yang Y., Zheng Z., Li Y., Liu Y., Yu L., Hua Z. (2019). *Apios americana* Medik flowers polysaccharide (AFP-2) attenuates H_2_O_2_ induced neurotoxicity in PC12 cells. Int. J. Biol. Macromol..

[44] Tsai Y.R., Chang C.F., Lai J.H., Wu J.C.C., Chen Y.H., Kang S.J., Hoffer B.J., Tweedie D., Luo W., Greig N.H. (2018). Pomalidomide ameliorates H_2_O_2_-induced oxidative stress injury and cell death in rat primary cortical neuronal cultures by inducing anti-oxidative and anti-apoptosis effects. Int. J. Mol. Sci..

[45] Lu M.C., Zhao J., Liu Y.T., Liu T., Tao M.M., You Q.D., Jiang Z.Y. (2019). CPUY192018, a potent inhibitor of the Keap1-Nrf2 protein-protein interaction, alleviates renal inflammation in mice by restricting oxidative stress and NF-κB activation. Redox Biol..

[46] Ren B., Yuan T., Diao Z., Zhang C., Liu Z., Liu X. (2018). Protective effects of sesamol on systemic oxidative stress-induced cognitive impairments: Via regulation of Nrf2/Keap1 pathway. Food Funct..

[47] Zhao F., Liu C., Fang L., Lu H., Wang J., Gao Y., Gabbianelli R., Min W. (2021). Walnut-Derived Peptide Activates PINK1 via the NRF2/KEAP1/HO-1 Pathway, Promotes Mitophagy, and Alleviates Learning and Memory Impairments in a Mice Model. J. Agric. Food Chem..

[48] Mustafa O., Gu K., Esra C. (2016). Antioxidant Activity/Capacity Measurement. 1. *Classi fication*, Physicochemical Principles, Mechanisms, and Electron Transfer (ET)-Based Assays. J. Agric. Food Chem..

[49] Li L., Liu J., Nie S., Ding L., Wang L., Liu J., Liu W., Zhang T. (2017). Direct inhibition of Keap1–Nrf2 interaction by egg-derived peptides DKK and DDW revealed by molecular docking and fluorescence polarization. RSC Adv..

[50] Wang S., Su G., Zhang X., Song G., Zhang L., Zheng L., Zhao M. (2021). Characterization and Exploration of Potential Neuroprotective Peptides in Walnut (*Juglans regia*) Protein Hydrolysate against Cholinergic System Damage and Oxidative Stress in Scopolamine-Induced Cognitive and Memory Impairment Mice and Zebrafish. J. Agric. Food Chem..

[51] Boiangiu R.S., Mihasan M., Gorgan D.L., Hritcu L., Stache B.A. (2021). Antioxidant Effects of Cotinine and 6-Hydroxy-L-Nicotine in Scopolamine-Induced Zebrafish (*Danio rerio*) Model of Alzheimer’s Disease. Antioxidants.

[52] Coradini K., de-Andrade D.F., Altenhofen S., Reolon G.K., Nery L.R., Silva N.E., Roca-Vianna M.R.M., Bonan C.D., Beck R.E.R. (2021). Free and nanoencapsulated curcumin prevents scopolamine-induced cognitive impairment in adult zebrafish. J. Drug Deliv. Sci. Technol..

[53] Willcox D.C., Scapagnini G., Willcox B.J., Science H. (2014). Healthy aging diets other than the Mediterranean: A Focus on the Okinawan Diet. Mech. Ageing Dev..

[54] Schliebs R., Arendt T. (2011). The cholinergic system in aging and neuronal degeneration. Behav. Brain Res..

[55] Schliebs R., Arendt T. (2006). The significance of the cholinergic system in the brain during aging and in Alzheimer’s disease. J. Neural Transm..

[56] Singh M., Kaur M., Kukreja H., Chugh R., Silakari O., Singh D. (2013). Acetylcholinesterase inhibitors as Alzheimer therapy: From nerve toxins to neuroprotection. Eur. J. Med. Chem..

[57] Sharma K. (2019). Cholinesterase inhibitors as Alzheimer’s therapeutics (Review). Mol. Med. Rep..

[58] Capatina L., Boiangiu R.S., Dumitru G., Napoli E.M., Ruberto G., Hritcu L. (2020). Todirascu-Ciornea E Rosmarinus officinalis Essential Oil Improves Scopolamine-Induced Neurobehavioral Changes via Restoration of Cholinergic Function and Brain Antioxidant Status in Zebrafish (*Danio rerio*). Antioxidants.

[59] Salim S. (2017). Oxidative Stress and the Central Nervous System. J. Pharmacol. Exp. Ther..

[60] Tu S., Okamoto S., Lipton S.A., Xu H. (2014). Oligomeric Aβ-induced synaptic dysfunction in Alzheimer’s disease. Mol. Neurodegener..

[61] Butterfield D.A., Boyd-kimball D. (2018). Oxidative Stress, Amyloid-β Peptide, and Altered Key Molecular Pathways in the Pathogenesis and Progression of Alzheimer’s Disease. J. Alzheimer’s Dis..

[62] Wang S., Zheng L., Zhao T., Zhang Q., Liu Y., Sun B., Su G., Zhao M. (2020). Inhibitory Effects of Walnut (*Juglans regia*) Peptides on Neuroin fl ammation and Oxidative Stress in Lipopolysaccharide-Induced Cognitive Impairment Mice. J. Agric. Food Chem..

[63] Prabha N., Guru A., Harikrishnan R., Gatashesh M., Hataleh A.A., Juliet A., Arockiaraj J. (2022). Neuroprotective and antioxidant capability of RW20 peptide from histone acetyltransferases caused by oxidative stress-induced neurotoxicity in in vivo zebrafish larval model. J. King Saud Univ.-Sci..

[64] Zheng L., Zhao Y., Dong H., Su G., Zhao M. (2016). Structure–activity relationship of antioxidant dipeptides: Dominant role of Tyr, Trp, Cys and Met residues. J. Funct. Foods.

[65] Han Z., Shen F., He Y., Degos V., Camus M., Maze M., Young W., Su H. (2014). Activation of a-7 Nicotinic Acetylcholine Receptor Reduces Ischemic Stroke Injury through Reduction of Pro-Inflammatory Macrophages and Oxidative Stress. PLoS ONE.

[66] Vo T., Ryu B., Kim S. (2013). Purification of novel anti-inflammatory peptides from enzymatic hydrolysate of the edible microalgal Spirulina maxima. J. Funct. Foods.

[67] Kim E., Kim Y.S., Hwang J.W., Kang S.E., Choi D.K., Lee K.H., Lee J.S., Moon S.H., Jeon B.T., Park P.J. (2013). Purification of a novel nitric oxide inhibitory peptide derived from enzymatic hydrolysates of *Mytilus coruscus*. Fish Shellfish Immunol..

[68] Lee S., Kim E.K., Kim Y.S., Hwang J.W., Lee K.H., Choi D.K., Kang H., Moon S.H., Jeon B.T., Park P.J. (2012). Purification and characterization of a nitric oxide inhibitory peptide from *Ruditapes philippinarum*. Food Chem. Toxicol..

[69] Hwang J., Lee S.J., Kim Y.S., Kim E.K., Ahn C.B., Jeon Y.J., Moon S.H., Jeon B.T., Park P.J. (2012). Purification and characterization of a novel peptide with inhibitory effects on colitis induced mice by dextran sulfate sodium from enzymatic hydrolysates of *Crassostrea gigas*. Fish Shellfish Immunol..

[70] Bamdad F., Bark S., Kwon C.H., Suh J.W., Sunwoo H. (2017). Anti-Inflammatory and Antioxidant Properties of Peptides Released from β-Lactoglobulin by High Hydrostatic Pressure-Assisted Enzymatic Hydrolysis. Molecules.

[71] Bellezza I., Giambanco I., Minelli A., Donato R. (2018). Nrf2-Keap1 signaling in oxidative and reductive stress. BBA-Mol. Cell Res..

[72] Kaspar J.W., Niture S.K., Jaiswal A.K. (2009). Nrf2: INrf2 (Keap1) signaling in oxidative stress. Free Radic. Biol. Med..

[73] Peng S., Wuu J., Mufson E.J., Fahnestock M. (2005). Precursor form of brain-derived neurotrophic factor and mature brain-derived neurotrophic factor are decreased in the pre-clinical stages of Alzheimer’s disease. J. Neurochem..

[74] Barco A., Bailey C.H., Kandel E.R. (2006). Common molecular mechanisms in explicit and implicit memory. J. Neurochem..

[75] Lesuis S.L., Hoeijmakers L., Korosi A., de Rooij S.R., Swaab D.S., Kessels H.W., Lucassen P.J., Krugers H.J. (2018). Vulnerability and resilience to Alzheimer’s disease: Early life conditions modulate neuropathology and determine cognitive reserve. Alzheimer’s Res. Ther..

[76] Neurochemistry J.O.F. (2018). Transcriptional co-repressor SIN3A silencing rescues decline in memory consolidation during scopolamine-induced amnesia. J. Neurochem..

[77] Lai S., Chen J.H., Lin H.Y., Liu Y.S., Tsai C.F., Chang P.C., Lu D.Y., Lin C. (2018). Regulatory Effects of Neuroinflammatory Responses through Brain-Derived Neurotrophic Factor Signaling in Microglial Cells. Mol. Neurobiol..

