# Supplementary material for: Characterization and Exploration of the Neuroprotective Potential of Oat-Protein-Derived Peptides in PC12 Cells and Scopolamine-Treated Zebrafish
Source: Nutrients. 2023 Dec 29;16(1):117. doi: 10.3390/nu16010117 (PMC10780882; doi:10.3390/nu16010117)
Supplement: Supplementary file 1 [file nutrients-16-00117-s001.zip › nutrients-2762759-supplementary.pdf]

Supplementary Materials

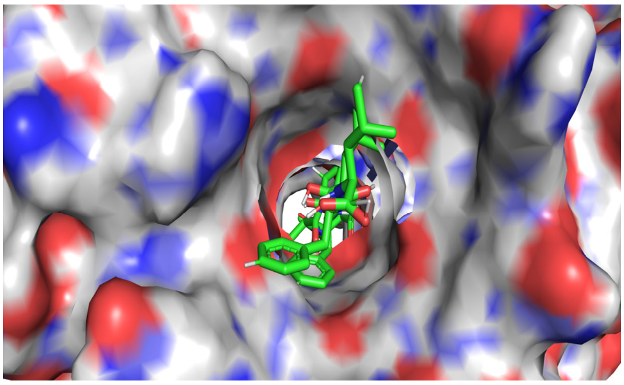

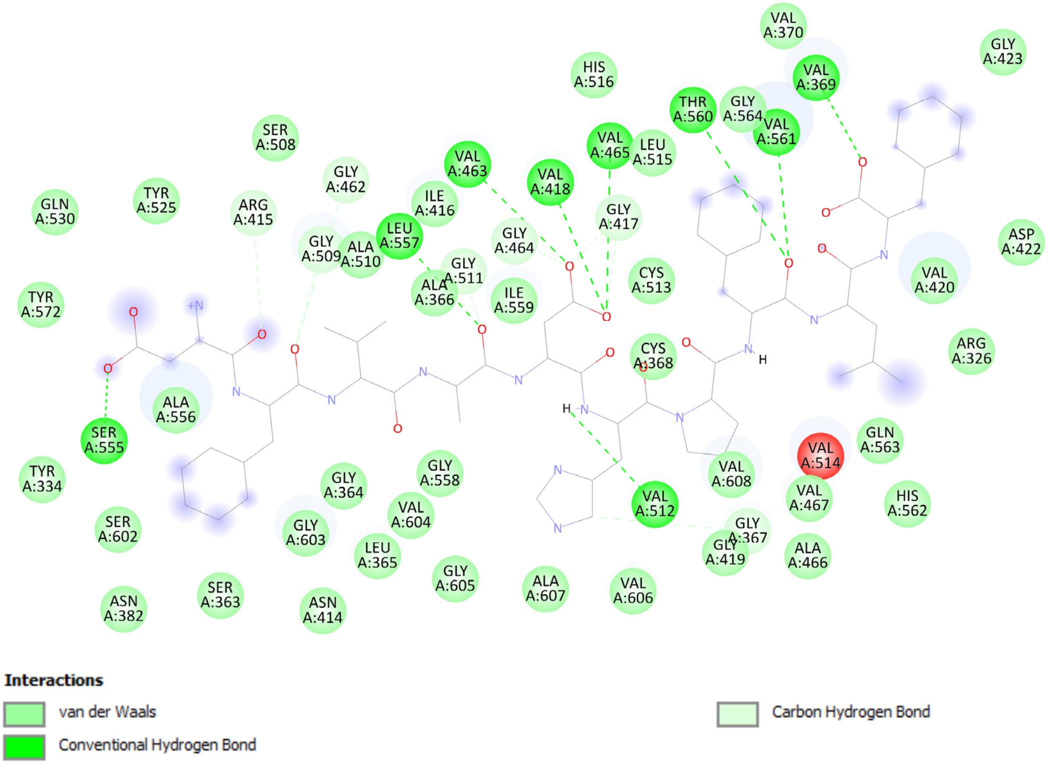

(DFVADHPFLF)

Supplementary Figure S1A

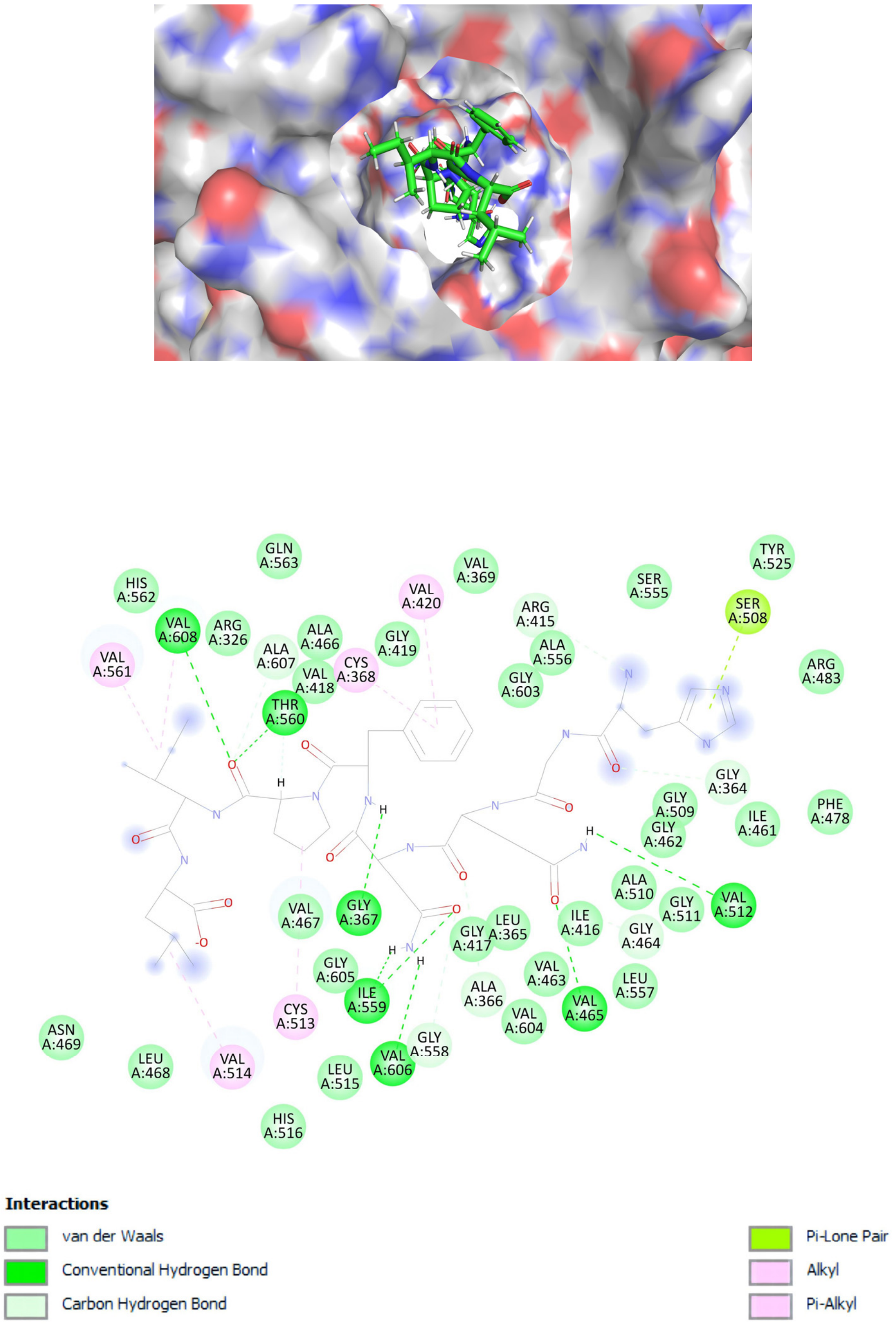

(HGQNFPIL)

Supplementary Figure S1B

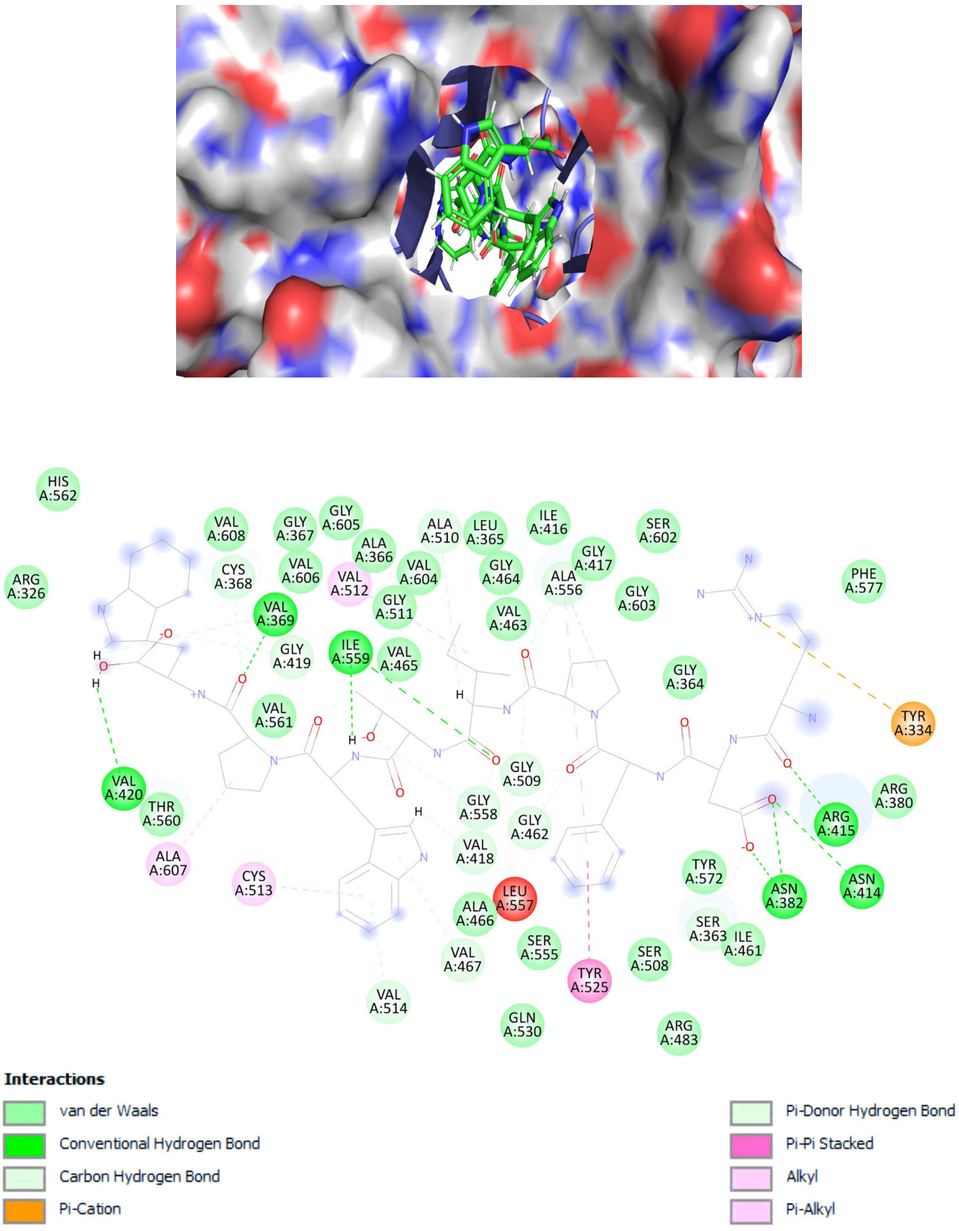

(RDFPITWPW)

Supplementary Figure S1C

Supplementary Figure S1. Molecular-docking interaction of peptides with Keap1. (A) DFVADHPFLF interaction with Keap1; (B) HGQNFPIL interaction with Keap1; (C) RDFPITWPW interaction with Keap1

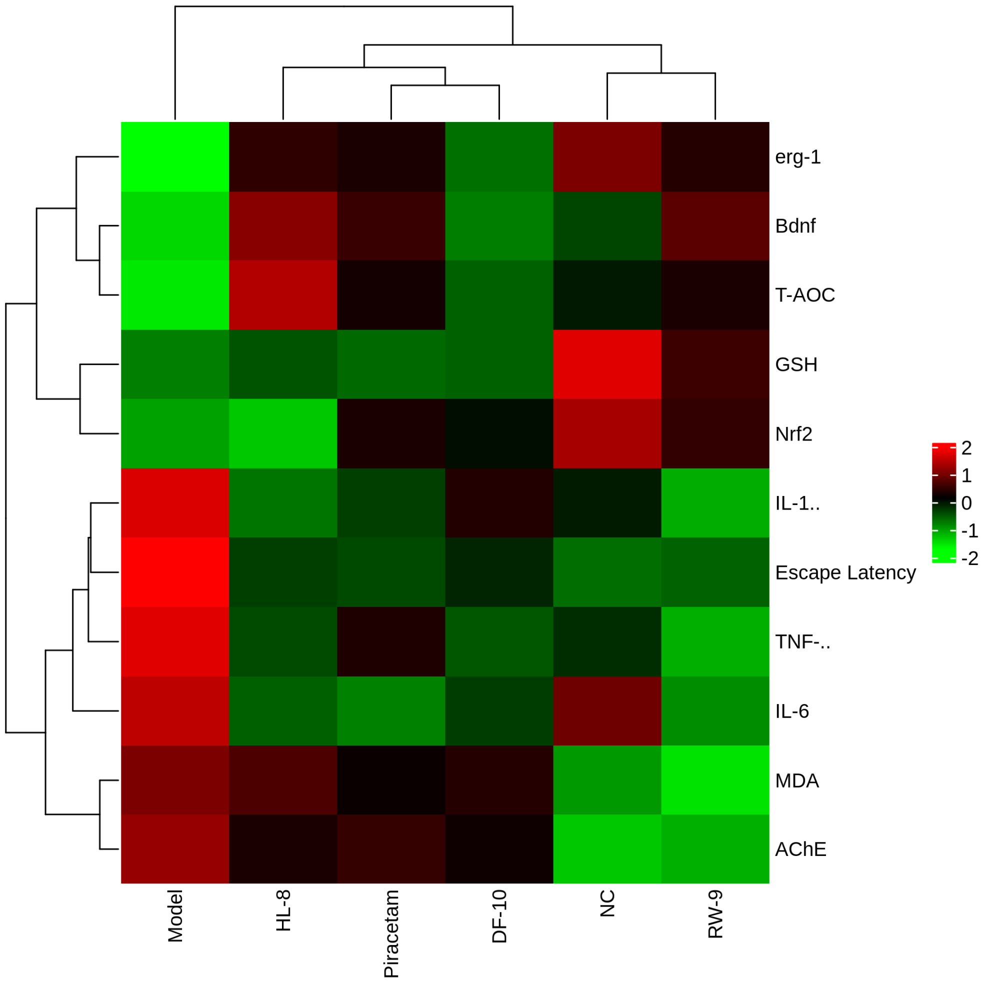

Supplementary Figure S2. Heatmap showing the results of a correlation analysis of samples and biochemical parameters

Supplementary Table S1. Primer sequences for RT-qPCR analysis

	**forward primers**	**reverse primers**
Nrf2	5-TCCTCTGCTGCCATTAGTC-3	5-TGCCTTCAGTGTGCTTCT-3
Keap1	5-CTCAACCGCTTGCTGTAT	5-TAATCATCCGCCACTCATTC
HO-1	5-GGTGACAGAAGAGGCTAA-3	5-GCAGAGGTAGTATCTTGAAC-3
β-actin	5-CTATCGGCAATGAGCGGTTCC-3	5-TGTGTTGGCATAGAGGTCTTTACG-3
Nrf2a	5′-ATG TCT AAA ATG CAG CCA AGC C-3′	5′-CGG TAG CTG AAG TCG AAC AC-3′
Bdnf	5′-GCT CTC TCA ATG CGC ACT AC-3′	5′-TGA CTG AGC GGA TCC TTT GG-3′
Egr1	5′-AGT TTG ATC ACC TTG CTG GAG-3′	5′-AAC GGC CTG TGT AAG ATA TGG-3′
GAPDH	5′-CCTTGTGAGCGCAGTAACTCT-3′	5′-TCCTTGTGGGGTCAGTCCTT-3′

Supplementary Table S2: Characteristics of peptides isolated from OPH and identified by LC-MS/MS

No#	Sequence	Length	Mass (Da)	Identification Score	Peak Intensity
1	AAGRVPGYGVLT	12	1159.635	55.112	171060000
2	ADHPFLFL	8	958.49125	54.525	39672000
3	AFRVPTVDVS	10	1089.5819	83.265	709910000
4	AGNNKREQQFGQ	12	1375.6593	135.43	30967000
5	AGNNKREQQFGQN	13	1489.7022	70.056	49507000
6	AGNNKREQQSGNNIF	15	1675.8026	110.08	71879000
7	AILSPFWNINAH	12	1381.7143	136.16	92805000
8	AIRGVYDGSLF	11	1196.619	68.224	110230000
9	AKIINDNFGIV	11	1202.6659	64.42	954490000
10	AKVINDKF	8	933.52837	92.187	193970000
11	AKVINDKFGIVEGL	14	1501.8504	72.096	123260000
12	ALPGGGVR	8	725.41842	83.287	165870000
13	ANQLEPRQK	9	1082.5833	98.997	10113000
14	ANQLEPRQKEFLLA	14	1655.8995	94.584	0
15	APLAKVIN	8	824.51199	71.949	4020500000
16	APSKDAPMF	9	962.45315	69.198	455300000
17	APSKDAPMFVM	11	1192.5621	76.827	1559200000
18	APSKDAPMFVV	11	1160.59	78.149	1584500000
19	APSKDAPMFVV	12	1217.6114	77.677	530090000
20	AQIPEQVR	8	939.51378	67.344	885900000
21	AQRDFARILPTPR	13	1539.8634	84.753	99999000
22	ARKNIENPQHADTY	14	1655.8016	48.532	20075000
23	ARQNIENPKRA	11	1295.7058	150.61	32758000
24	ARQNIENPKRADTYNPR	17	2042.0406	164.01	31469000
25	ATRVNLYQNAILSPF	15	1705.9152	118.77	321850000
26	ATRVNLYQNAILSPY	15	1721.9101	93.839	74876000
27	AVEHELGGF	9	957.4556	73.039	224420000
28	AVEHELGGFL	10	1070.5397	69.176	2562900000
29	AVGKVLPVLN	10	1008.6332	74.127	807970000
30	AVGKVLPVLNGK	12	1193.7496	80.676	28874000
31	AVNLIPFPR	9	1025.6022	148.07	357550000

32	AVNLIPFPRLH	11	1275.7452	124.08	368330000
33	AVPFLRSQILRQ	12	1426.8409	109.15	443260000
34	AVQGELGGFL	10	989.5182	62.717	121890000
35	AYQPIQSQ	8	933.4556	135.87	1491500000
36	DFARILPTPR	10	1184.6666	138.08	354260000
37	DFGWGRPVFM	10	1210.5593	62.546	44557000
38	DFVADHPFLF	10	1206.571	67.169	1587200000
39	DFVADHPFLFL	11	1319.655	73.248	618680000
40	DHHDRFMPF	9	1200.5135	125.58	444130000
41	DILRRGQLL	9	1082.656	111.04	154340000
42	DMSTPEFPSGR	11	1222.5288	81.972	47693000
43	DQNGDGIIYPW	11	1276.5724	75.825	261780000
44	DRLRQGQLL	9	1097.6305	86.794	304890000
45	DTEGPPFMAR	10	1119.5019	139.68	490650000
46	DTEGRFMPVNFENIFSK	17	2029.9568	126.56	555030000
47	DTYNPRAGRIT	11	1262.6367	83.397	133980000
48	DWYKGPTL	8	978.48108	77.741	71967000
49	DWYKGPTLL	9	1091.5651	75.692	2178700000
50	DWYKGPTLLE	10	1220.6077	73.573	79996000
51	EDVSGVVL	8	816.4229	72.355	175770000
52	EHELGGFLGL	10	1070.5397	71.296	2562900000
53	EQYQPYPEQQQPFLQ	15	1921.8846	73.632	4308600000
54	EQYQPYPEQQQPIL	14	1759.8417	61.213	443160000
55	EQYQPYPEQQQPILQQ	16	2015.9589	84.884	188850000
56	ESHVADRIGYY	11	1308.6099	136.21	134050000
57	EVPFLRSQIL	10	1200.6867	90.37	208740000
58	FAGDDAPRAVFPS	13	1348.6412	88.596	59965000
59	FDPSEQYQPYPEQQQPF	17	2126.9222	45.911	42177000
60	FDPSEQYQPYPEQQQPFLQ	19	2368.0648	53.034	57755000
61	FDQNGDGIIYPW	12	1423.6408	72.607	336380000
62	FFDQNGDGIIYPW	13	1570.7092	60.019	471750000
63	FIDNIFRF	8	1070.5549	90.986	53257000
64	FIGRRSRPDQIGGL	14	1570.8692	123.42	2457900000
65	FLKPIVSQ	8	930.55385	79.469	12889000000
66	FNDILRRG	8	989.54066	80.098	129180000
67	FNDILRRGQLL	11	1343.7674	152.11	1170500000
68	FNDRLRQGQLL	11	1358.7419	120.12	399770000
69	FNPDKSPAYPIRF	13	1550.7882	133.99	1791200000
70	FPGIADRM	8	905.44292	107.32	248070000
71	FRVPTVDVS	9	1018.5447	65.252	422430000
72	FRVPTVDVSV	10	1117.6132	86.67	71537000
73	FSLAPLVPRL	10	1111.6754	136.11	51651000
74	FSYRDNPRDIQLN	13	1636.7958	162.38	390060000

75	FVADHPFLFL	10	1204.6281	64.236	311180000
76	GAELIDSVLDVVRK	14	1512.8512	133.89	249340000
77	GAPSAPIEKL	10	981.5495	60.91	224520000
78	GDGIIYPWETFRGL	14	1622.8093	53.08	47366000
79	GFAGDDAPRAVFPS	14	1405.6626	91.313	134650000
80	GFDTEGPPFMAR	12	1323.5918	161.79	627680000
81	GIGTVPVGR	9	854.4974	128.57	512560000
82	GIGTVPVGRVE	11	1082.6084	113.62	3319000000
83	GIGTVPVGRVETG	13	1240.6776	112.75	1322200000
84	GIGTVPVGRVETGVIKPG	18	1734.9992	110.9	1488200000
85	GISQQAAQRIQ	11	1198.6418	72.968	762730000
86	GIVHWGYNDGDAPVVAIY	18	1944.937	87.667	196580000
87	GLIGKRLKIA	10	1067.7179	96.285	220400000
88	GLPSRPLY	8	901.50215	82.263	644990000
89	GLQFLKPIVSQ	11	1228.718	95.358	361090000
90	GLQKGEIGKR	10	1084.6353	113.41	34046000
91	GLQKGEIGKRL	11	1197.7194	187.78	408230000
92	GLQKGEIGKRLL	12	1310.8034	176.6	121850000
93	GLQKGEIGKRLLR	13	1466.9045	97.214	647510000
94	GLQKGEIGKRLLRAK	15	1666.0366	191.04	420240000
95	GLSYPTLPTWRPS	13	1473.7616	123.79	112610000
96	GNNKEDQQFGQ	11	1263.548	147.36	63616000
97	GNNKEDQQFGQN	12	1377.5909	111.06	105050000
98	GNNKEDQQFGQNIFSGF	17	1928.8653	150.46	57190000
99	GNNKREQQF	9	1119.5421	65.246	7141500
100	GNNKREQQFGQ	11	1304.6222	121.8	243610000
101	GNNKREQQFGQN	12	1418.6651	153.97	401110000
102	GNNKREQQFGQNIF	14	1678.8176	96.009	140300000
103	GNNKREQQFGQNIFSGF	17	1969.9395	157.99	490240000
104	GNNKREQQSGNNIF	14	1604.7655	152.67	1587800000
105	GQGEQDRFVLK	11	1275.6571	115.06	67830000
106	GQTVFNDILRR	11	1317.7153	155.42	299860000
107	GQTVFNDILRRGQLL	15	1728.9635	163.12	4628900000
108	GQTVFNDILRRGQLLIIPQ	19	2180.243	189.61	297110000
109	GQTVFNDRLRQGQLL	15	1743.938	141.08	249490000
110	GRVPGYGVLT	10	1017.5607	86.794	326450000
111	GVEPVIGVR	9	924.53927	107.67	385210000
112	GVFTDKDKAAAH	12	1258.6306	68.069	98451000
113	GVNEKEYTSDITIV	14	1566.7777	113.1	48961000
114	GVRNPEEIPWA	11	1266.6357	83.313	134470000
115	GVRNPEEIPWAE	12	1395.6783	124.74	836720000
116	GVRNPEEIPWAEA	13	1466.7154	90.149	336120000
117	GVRNPEEIPWAEAG	14	1523.7369	78.488	1174000000

118	GWVANKGEWILL	12	1384.7503	95.909	123070000
119	HGQNFPIL	8	924.48175	52.693	1481800000
120	HIKQGDVV	8	894.49232	75.269	102500000
121	HLKDEHQRVH	10	1297.664	116.25	25354000
122	HNAPGLVY	8	869.43955	74.717	299080000
123	HQAYQPIQSQ	10	1198.5731	123.98	654110000
124	HVSFFDQN	8	992.43519	69.432	182210000
125	HVSFFDQNGDGIIYPW	16	1893.8686	227.94	271470000
126	HWGYNDGDAPVVA	13	1399.6157	134.77	52234000
127	IDAPGHRDFIKN	12	1381.7102	105.4	75842000
128	IDFVADHPFLF	11	1319.655	110.67	2227300000
129	IDFVADHPFLFL	12	1432.7391	88.819	439900000
130	IDSVLDVVRK	10	1142.6659	154.12	141740000
131	IENPKRAD	8	941.49304	102.72	8167000
132	IENPKRADTY	10	1205.6041	132.76	107990000
133	IENPKRADTYNPR	13	1572.8009	177.83	1520400000
134	IENPKRADTYNPRAGRIT	18	2071.0923	89.846	1017000000
135	IENPQHADTYNPR	13	1553.7223	175.15	162470000
136	ILRALPVDVL	10	1107.7016	91.626	97506000
137	IQRELGGFF	9	1065.5607	123.98	667800000
138	IREDVSGVVL	10	1085.6081	110.41	1579600000
139	IRRVIEPQ	8	1009.6033	126.28	340550000
140	IVRDIKEKL	9	1112.6917	120.87	306800000
141	KAERERGITIDIA	13	1470.8154	108.3	153200000
142	KDEHQRVH	8	1047.521	150.88	155970000
143	KDFPLTWPW	9	1188.5968	68.383	131770000
144	KDIPITWPW	9	1154.6124	84.568	2892200000
145	KDLLPVGGDFLL	12	1285.7282	92.538	191180000
146	KEDQQFGQNIFSGF	14	1643.758	112.11	42243000
147	KEQRGEII	8	971.53999	106.94	1540800000
148	KEQRGEIIR	9	1127.6411	88.136	96560000
149	KEQRGEIIRVT	11	1327.7572	103.34	462320000
150	KFIPELIGK	9	1043.6379	120.87	1047600000
151	KHGSDTATF	9	962.44576	67.981	900380
152	KIINDNFGIV	10	1131.6288	71.491	114110000
153	KIINDNFGIVE	11	1260.6714	117.81	495850000
154	KLPYHQGGDKRKF	13	1572.8525	119.6	990910000
155	KNIENPQHADTY	12	1428.6634	133.76	32752000
156	KNIENPQHADTYNPR	15	1795.8602	160.46	193490000
157	KNNRGEEF	8	992.46756	75.243	27107000
158	KNNRGEEFDAF	11	1325.6	111.06	425110000
159	KNNRGEEFGAF	11	1267.5945	120.31	849000000
160	KNNRGQESGVFTPK	14	1560.8009	169.58	922690000

161	KQGDVIALPA	10	1010.576	65.156	6617500000
162	KQGDVVAL	8	828.47052	51.787	0
163	KQGDVVALPA	10	996.5604	98.904	155550000
164	KREQQSGNNIF	11	1319.6582	78.516	47510000
165	KSYELPDGQVITI	13	1461.7715	85.807	195270000
166	KSYELPDGQVITIG	14	1518.793	72.096	916930000
167	KTRENMFHL	9	1174.5917	77.282	176860000
168	KVVEDNLGKIK	11	1241.7343	148.73	180210000
169	LENVTEEDAKATNL	14	1545.7522	51.268	37736000
170	LFPVYIDR	8	1021.5597	79.469	219520000
171	LIDSVLDVVRK	11	1255.75	87.258	175270000
172	LIEDVLPQ	8	925.51205	98.415	136380000
173	LIPFPRLH	8	991.59672	77.72	149570000
174	LKDEHQRVH	9	1160.6051	70.919	15086000
175	LLFPVYIDR	9	1134.6437	160.75	325280000
176	LLFPVYIDRIHKA	13	1583.9188	56.087	20712000
177	LQFLKPIV	8	956.60589	75.915	199840000
178	LQFLKPIVSQ	10	1171.6965	104.01	5554100000
179	LRALPIDV	8	895.5491	62.714	42039000
180	LRALPIDVL	9	1008.6332	116.54	321390000
181	LRALPVDVL	9	994.61752	104.45	1072900000
182	LRALPVDVLAN	11	1179.6976	85.67	0
183	LRSQILRQ	8	1012.6142	99.755	105750000
184	LSAISPNSMVSH	12	1241.6074	40.002	0
185	LSRGLIDL	8	885.52837	67.032	41969000
186	LSRGLIDLN	9	999.57129	61.962	273040000
187	LSYPTLPTWRPS	12	1416.7402	74.944	72061000
188	LVPYPRIH	8	993.57599	91.093	73722000
189	MIQGHARVQ	9	1038.5393	102.71	21503000
190	MQQQFFQPQ	9	1180.5335	83.614	72382000
191	MRDFPITWPW	10	1347.6434	127.56	2049700000
192	MTPQSPKPS	9	971.47462	83.265	8648300
193	MVPFLRSQ	8	976.51642	78.178	313480000
194	MVPFLRSQIL	10	1202.6845	80.229	471050000
195	MVPFLRSQILRQ	12	1486.8442	56.205	69277000
196	NARSQPDKL	9	1027.5411	106.38	18302000
197	NARSQPDKLTLREIWMM	17	2088.0608	87.388	92527000
198	NDILRRGQLL	10	1196.699	171.53	6036800000
199	NDILRRGQLLIIPQ	14	1647.9784	142.08	62056000
200	NDILRRGQLLIVPQ	14	1633.9628	72.891	33019000
201	NDQRGEII	8	943.47231	121.67	1618100000
202	NDQRGEIIR	9	1099.5734	147.24	143030000
203	NDQRGEIIRV	10	1198.6418	88.392	140770000

204	NDQRGEIIRVS	11	1285.6739	119.45	1707200000
205	NDRLRQGQL	9	1098.5894	93.429	176230000
206	NDRLRQGQLL	10	1211.6735	136.21	31666000000
207	NDRLRQGQLLI	11	1324.7575	89.886	162060000
208	NDRLRQGQLLIVPQ	14	1648.9373	157.25	147420000
209	NFENIFSK	8	997.4869	78.324	371370000
210	NFPILNLVQM	10	1187.6373	71.296	742300000
211	NFPILNLVQMS	11	1274.6693	58.119	267740000
212	NHGQTVFNDILRRGQLL	17	1980.0653	263.57	651490000
213	NHPGQIGNGY	10	1055.4785	111.65	426900000
214	NIENPKRA	8	940.50903	80.462	33477000
215	NIENPKRAD	9	1055.536	73.26	3394100
216	NIENPKRADTY	11	1319.647	121.64	45682000
217	NIENPKRADTYNPR	14	1686.8438	208.63	613610000
218	NIENPKRADTYNPRAGRIT	19	2185.1352	93.753	262400000
219	NKEDQQFGQ	9	1092.4836	125.5	7584400
220	NKEDQQFGQN	10	1206.5265	102	15698000
221	NKGEWILL	8	971.54402	108.98	2055800000
222	NKREQQFGQ	9	1133.5578	101.43	14641000
223	NKREQQFGQN	10	1247.6007	70.256	39615000
224	NKREQQFGQNIFSGF	15	1798.8751	100.69	48695000
225	NKREQQSGNNIF	12	1433.7011	122.96	205000000
226	NKTPGQVL	8	855.48142	58.167	373940000
227	NKTPGQVLIK	10	1096.6604	86.136	144250000
228	NLDLVVPR	8	924.53927	107.43	88697000
229	NLDWYKGPTLL	11	1318.6921	85.716	1582300000
230	NLIPFPRL	8	968.58074	77.496	34329000
231	NLIPFPRLH	9	1105.6396	97.071	367550000
232	NLKDEHQRVH	10	1274.648	149.82	516700000
233	NLKNNRGEEFGAF	13	1494.7215	91.656	147950000
234	NLKNNRGQESGVF	13	1461.7324	78.61	58722000
235	NLKNNRGQESGVFTPK	16	1787.9278	53.3	66241000
236	NLPSRSDVVQLY	12	1389.7252	76.345	42725000
237	NNANQLEPR	9	1054.5156	164.72	52613000
238	NNANQLEPRQKEFLLA	16	1883.9854	138.51	97240000
239	NNGQTVFNDILRRGQLL	17	1957.0494	191.29	437350000
240	NNGQTVFNDRLRQGQLL	17	1972.0239	80.387	48678000
241	NNHGQTVFNDILRRGQLL	18	2094.1083	48.899	98362000
242	NNKREQQFGQ	10	1247.6007	69.825	3712700
243	NPRAGRIT	8	883.4988	65.235	138260000
244	NSKNFPILN	9	1045.5556	83.206	839760000
245	NSKNFPILNI	10	1158.6397	75.043	99485000
246	NSKNFPILNIV	11	1257.7081	106.6	111580000

247	NSKNFPILNIVQM	13	1516.8072	91.265	138310000
248	NSKNFPTL	8	919.47633	71.141	6284000000
249	NSKNFPTLN	9	1033.5193	109.01	4780000000
250	NSKNFPTLNLVQM	13	1504.7708	103.26	579630000
251	NSKNFPTLNLVQMS	14	1591.8028	89.171	184150000
252	NWDDMEKIWHH	11	1509.6459	109.44	87357000
253	PFMQQKQPFMQQQQ	14	1792.8389	45.997	864790000
254	PFMQQQQQPFMQQK	14	1792.8389	46.891	864790000
255	PGGGVRLDPGKSWAL	15	1508.81	75.652	121070000
256	PSEQYQPYPEQQEPF	15	1865.8108	101.82	179420000
257	PTLPTWRP	8	966.5287	134.06	130030000
258	PTLPTWRPS	9	1053.5607	147.19	545530000
259	PTLPTWRPSL	10	1166.6448	96.948	146550000
260	QDRSFNGL	8	935.44609	66.429	103930000
261	QELREIAG	8	914.48214	62.464	21255000
262	QEQPFVQQ	8	1002.4771	99.919	157300000
263	QGLIGKRL	8	883.56034	80.462	15689000
264	QGLQFLKPF	9	1076.6019	65.803	939270000
265	QGLQFLKPIVSQ	12	1356.7765	83.499	1277300000
266	QGRGFTGL	8	834.4348	83.377	4885300000
267	QGRGYTGL	8	850.42972	78.616	191330000
268	QHVSFFDQNGDGIIYPW	17	2021.9272	286.95	67907000
269	QKGEIGKRL	9	1027.6138	90.862	337720000
270	QKGEIGKRLL	10	1140.6979	82.241	576190000
271	QKGEIGKRLLR	11	1296.799	132.13	387540000
272	QKGEIGKRLLRAK	13	1495.9311	144.65	548730000
273	QLLQPQLQ	8	966.54983	81.381	163220000
274	QMFLQPLL	8	988.54157	71.359	233840000
275	QNAILSPFWNINAH	14	1623.8158	143.03	184200000
276	QNIENPKRA	9	1068.5676	126.67	142680000
277	QNIENPKRADTY	12	1447.7056	55.452	63571000
278	QNIENPKRADTYNPR	15	1814.9024	208.46	750060000
279	QNIENPKRADTYNPRAGRIT	20	2313.1938	145.71	327320000
280	QNLKDEHQRVH	11	1402.7066	56.359	7617800
281	QQGLIGKR	8	898.53485	99.919	113340000
282	QQGLIGKRLKIA	12	1323.8351	141.48	190650000
283	QQPPFVQQ	8	970.48723	94.163	2705000000
284	QQQFFQPQ	8	1049.493	100.82	786400000
285	QQQLLQPQ	8	981.52434	82.263	150270000
286	QQQLLQPQL	9	1094.6084	88.029	293910000
287	QQQLLQPQLQ	10	1222.667	113.89	122310000
288	QQQLNPCRQFLMQQ	14	1760.845	122.13	160290000
289	QQQMFLQPL	9	1131.5747	64.803	160630000

290	QQQQFFQPQ	9	1177.5516	110.36	265710000
291	QQQQFFQPQL	10	1290.6357	94.662	208160000
292	QQQQFIQPQ	9	1143.5673	110.61	215870000
293	QQQQFIQPQL	10	1256.6513	92.187	72937000
294	QQQQVVQAQQMGLVQPQTQ	19	2166.0851	43.544	144100000
295	QQQVFQPQ	8	1001.493	82.263	366820000
296	QQVFIPPQLQ	10	1196.6554	104.45	832420000
297	QQVFIPPQLQQ	11	1324.7139	86.794	231120000
298	QRELGGFF	8	952.47667	94.662	160110000
299	QRGTSVIPK	9	984.57163	70.525	69371000
300	QRLQFLKPT	9	1129.6608	126.67	5488000000
301	QRLQFLKPTM	10	1260.7013	124.83	284080000
302	QRLQFLKPTMS	11	1347.7333	99.442	73435000
303	QRPVRDDLE	9	1126.5731	90.37	216320000
304	QRPVRDDLEKHIPKPY	16	1990.0748	217.22	1187100000
305	QSQDRSFN	8	980.43117	93.195	44325000
306	QSQNDQRGEII	11	1286.6215	100.57	110170000
307	QSSRQGGLK	9	959.51484	92.47	9672500
308	QSSRQGGLR	9	987.52099	163.04	214840000
309	QVFIPPQLQ	9	1068.5968	117.16	6911200000
310	QVFIPPQLQQ	10	1196.6554	87.323	832420000
311	QVFNQPQMQ	9	1118.5179	85.359	223850000
312	QVFNQPQQQ	9	1115.536	86.872	354130000
313	QVGQSPQYQE	10	1162.5255	75.738	23930000
314	QVGQSPQYQEGQ	12	1347.6055	78.516	53847000
315	QVQQQVFQPQ	10	1228.62	67.981	17667000
316	QYQPYPEQQQPIL	13	1630.7991	68.846	67634000
317	QYQPYPEQQQPILQ	14	1758.8577	127.76	273860000
318	RADTYNPR	8	991.48354	154.71	11833000
319	RALPIDVL	8	895.5491	126.24	18795000000
320	RALPIDVLA	9	966.58622	62.659	697180000
321	RALPVDVL	8	881.53345	128.75	16397000000
322	RALPVDVLA	9	952.57057	73.616	685640000
323	RALPVDVLAN	10	1066.6135	123.98	7929500000
324	RAVEHELGGF	10	1113.5567	81.548	113840000
325	RAVEHELGGFL	11	1226.6408	101.25	2647900000
326	RAVQGELGGFL	11	1145.6193	72.23	0
327	RDFARILPTPR	11	1340.7677	115.7	9466800000
328	RDFPITWPW	9	1216.6029	107.66	3734900000
329	REIVRDIKEKL	11	1397.8354	76.85	103360000
330	RELIIGDR	8	970.55598	169.09	660730000
331	REQQFGQN	8	1005.4628	81.191	14651000
332	RFEEIVKE	8	1048.5553	70.69	69127000

333	RFEEIVKEVGSY	12	1454.7405	101.97	367340000
334	RFEEIVKEVGSYL	13	1567.8246	104.43	362690000
335	RFKQGDVIALPA	12	1313.7456	75.571	195400000
336	RGEEFGAF	8	911.41373	65.777	60171000
337	RGEIIRVS	8	928.54541	54.982	44955000
338	RGEIIRVT	8	942.56106	83.429	771060000
339	RGITIDIA	8	857.49707	76.847	606340000
340	RGQESGVFTPK	11	1204.62	89.886	33865000
341	RIIGLDNI	8	912.53927	103.29	1613800000
342	RLHGQNFPILNL	12	1420.7939	68.224	73648000
343	RLKDGAHQPPEAGEVL	16	1715.8955	106.26	306550000
344	RLQFLKPT	8	1001.6022	128.48	750160000
345	RLRQGQLL	8	982.6036	74.396	806350000
346	RQNIENPKRA	10	1224.6687	77.533	28657000
347	RQNIENPKRADTY	13	1603.8067	62.528	40119000
348	RQNIENPKRADTYNPR	16	1971.0035	119.54	127020000
349	RRSRPDQIGGL	11	1253.6953	79.489	551270000
350	RSAPAFIELDTK	12	1346.7194	61.65	79717000
351	RSRPDQIGGL	10	1097.5942	87.639	688860000
352	RSTNLDWYKGPT	12	1436.7048	76.143	63573000
353	RSTNLDWYKGPTLL	14	1662.873	121.61	1250600000
354	RSTNLDWYKGPTLLE	15	1791.9155	67.252	67686000
355	RTIDFVADHPFLF	13	1576.8038	126.31	389750000
356	RVAPEDHPVLL	11	1244.6877	80.165	342270000
357	RVAPEEHPVLL	11	1258.7034	87.476	303980000
358	RVAPEEHPVLLT	12	1359.751	56.013	48074000
359	RVNLYQNAILSPF	13	1533.8304	166.52	146560000
360	RVNLYQNAILSPY	13	1549.8253	128.38	25516000
361	RVPTVDVS	8	871.47633	95.793	3046000000
362	RVPTVDVSV	9	970.54475	85.212	161820000
363	SATRVNLY	8	922.48723	118.89	0
364	SATRVNLYQNAILSPF	16	1792.9472	155.48	238960000
365	SATRVNLYQNAILSPY	16	1808.9421	130.44	65946000
366	SFFDQNGDGIIYPW	14	1657.7413	86.898	114130000
367	SFHRVIPDFM	10	1247.6121	90.601	46036000
368	SFQPYPEGEDESSLTN	16	1798.7534	57.836	392030000
369	SFRVPTVDVS	10	1105.5768	73.985	2181000000
370	SGGQRDLFEGIL	12	1290.6568	83.358	84880000
371	SGVFTPKF	8	881.4647	66.993	1657900000
372	SHDVITGR	8	883.45118	151.56	77239000
373	SHLKDEHQRVH	11	1384.696	117.89	506560000
374	SHVADRIGYYK	11	1307.6622	164.85	528600000
375	SILRALPIDVL	11	1208.7493	82.645	246450000

376	SILRALPIDVLAN	13	1393.8293	108.7	459820000
377	SILRALPVDVL	11	1194.7336	83.948	637980000
378	SILRALPVDVLAN	13	1379.8136	82.261	233330000
379	SIQHELGGFF	10	1133.5506	65.465	847740000
380	SIQRELGGFF	10	1152.5928	123.35	356510000
381	SIQRELGGFFGT	12	1310.6619	86.014	72352000
382	SKNFPTLNLVQM	12	1390.7279	114.5	97248000
383	SLDNLKIL	8	914.54368	112.37	105120000
384	SMPRTIDFVADHPF	14	1631.7766	74.53	41877000
385	SMPRTIDFVADHPFLF	16	1891.9291	132.47	1647100000
386	SPVAAVPFL	9	899.51165	64.523	22440000
387	SPVAEVPFL	9	957.51713	92.191	1647100000
388	SPVAEVPFLR	10	1113.6182	183.65	6627500000
389	SPVAVVPF	8	814.45889	71.359	231640000
390	SPVAVVPFL	9	927.54295	104.11	432490000
391	SQKEQRGEIIR	11	1342.7317	80.834	84819000
392	SQKEQRGEIIRVT	13	1542.8478	84.821	1137800000
393	SQNDQRGEII	10	1158.5629	102.62	1127500000
394	SQNDQRGEIIR	11	1314.664	102.06	77275000
395	SQNDQRGEIIRV	12	1413.7324	93.561	46541000
396	SQNDQRGEIIRVS	13	1500.7645	176.87	930330000
397	SQNLKDEHQRVH	12	1489.7386	114.5	46653000
398	SQSHLKDEHQRVH	13	1599.7866	102.62	13972000
399	SQTIKDEHQRVQ	12	1467.743	96.342	16137000
400	SRIDHKFDLM	10	1260.6285	107.69	312630000
401	SRPDQIGGL	9	941.49304	91.853	1671300000
402	SSDTIDNVKAK	11	1176.5986	65.156	7380800
403	SSRQGGLR	8	859.46241	108.09	67867000
404	SSTPWQSSRQGGLR	14	1545.7648	115.89	236900000
405	STDKAAGRVPGY	12	1220.6149	85.733	214920000
406	STDKAAGRVPGYGVLT	16	1590.8366	130.77	351010000
407	STNLDWYKGPTLL	13	1506.7718	87.066	457120000
408	STPWQSSRQGGLR	13	1458.7328	116.75	228690000
409	SVMHMIQGRARVQ	13	1511.7813	63.727	267760000
410	SYPTLPTWRP	10	1216.6241	104.44	541750000
411	SYPTLPTWRPSL	12	1416.7402	109.29	1532700000
412	SYPTLPTWRPSLL	13	1529.8242	78.655	52944000
413	SYTVWPGALPGGGVR	15	1515.7834	123.63	70644000
414	TAGLIEDVLPQ	11	1154.6183	101.43	887720000
415	TFDTEGRFMPV	11	1298.5965	74.464	58833000
416	TGLSYPTLPTWRP	13	1487.7773	145.25	44242000
417	TGLSYPTLPTWRPS	14	1574.8093	107.06	313150000
418	TGLSYPTLPTWRPSL	15	1687.8934	105.03	126230000

419	TIDFVADHPFL	11	1273.6343	75.738	247100000
420	TIDFVADHPFLF	12	1420.7027	123.16	15573000000
421	TIDFVADHPFLFL	13	1533.7868	111.06	3382300000
422	TIKDEHQRVQ	10	1252.6524	118.74	130420000
423	TLREIWMM	8	1078.5304	77.923	312870000
424	TNLDWYKGPTLL	12	1419.7398	98.257	355220000
425	TSMPRTIDFVADHPFLF	17	1992.9768	101.56	186040000
426	TTGGWPTAPDGPY	13	1318.583	59.597	174370000
427	TTMRDFPITWPW	12	1549.7388	121.8	3964400000
428	TVFNDILR	8	976.53418	123.69	643840000
429	TVFNDILRR	9	1132.6353	122.19	4447200000
430	TVFNDILRRG	10	1189.6568	121.05	616950000
431	TVFNDILRRGQL	12	1430.7994	70.488	41253000
432	TVFNDILRRGQLL	13	1543.8835	143.96	3409800000
433	TVFNDRLRQG	10	1204.6313	90.108	713830000
434	TVFNDRLRQGQLL	13	1558.858	117.8	1072500000
435	TVIDAPGHRDFIK	13	1467.7834	149.33	371470000
436	TVIDAPGHRDFIKN	14	1581.8263	136.94	529890000
437	TVPVGRVETGVIKPG	15	1507.8722	61.235	0
438	VA**DHPFLFL**	9	1057.5597	72.355	370880000
439	VADRIGYY	8	955.47633	81.191	382220000
440	VAPFLRSQ	8	916.51305	94.539	234720000
441	VAPFLRSQIL	10	1142.6812	62.659	408510000
442	VDVNNPEGTKGGRH	14	1478.7226	70.977	3211700
443	VFDVNNNANQLEPRQKEFLLA	21	2458.2605	182.02	424730000
444	VFIPPQLQ	8	940.5382	138.38	28108000000
445	VFIPPQLQQ	9	1068.5968	95.793	5457300000
446	VFNDILRR	8	1031.5876	79.116	224100000
447	VFNDILRRG	9	1088.6091	66.621	64355000
448	VFNDILRRGQLL	12	1442.8358	116.12	1197000000
449	VFNDRLRQGQLL	12	1457.8103	119.75	232150000
450	VFNQPQMQ	8	990.4593	68.485	81310000
451	VFNQPQQQ	8	987.47739	74.717	135640000
452	VIDAPGHR	8	863.46135	124.37	0
453	VIDAPGHRDFIK	12	1366.7357	140.78	363670000
454	VIDAPGHRDFIKN	13	1480.7787	104.82	397640000
455	VIGSPLVELLR	11	1194.7336	72.434	73541000
456	VIISAPSKDAPMFVM	15	1604.8306	65.933	242000000
457	VINDKFGIVEGL	12	1302.7184	71.614	46547000
458	VIRRVIEPQ	9	1108.6717	127.12	4978100000
459	VNLIPFPRLH	10	1204.7081	64.485	95933000
460	VPFLRSQILRQ	11	1355.8038	61.344	57285000
461	VQKFIPELIGK	11	1270.7649	82.914	80161000

462	VREQYMVK	8	1051.5485	121.99	302480000
463	VSQQGPVEHQ	10	1107.5309	63.419	4084200
464	VVAPPERKYSVW	12	1429.7718	90.696	69401000
465	VVISAPSKDAPMF	13	1360.7061	70.056	70864000
466	VVLYPSPGMGHL	12	1268.6587	116.54	202390000
467	VVNNHGQTVF	10	1113.5567	70.69	181230000
468	VVNNHGQTVFNDILRRGQLL	20	2292.2451	229.97	2894100000
469	VVNNHGQTVFNDILRRGQLLIIPQ	24	2743.5246	79.69	71855000
470	VVNNNGQTVFNDILRR	16	1857.9809	112.72	62939000
471	VVNNNGQTVFNDILRRGQLL	20	2269.2291	258.23	4093900000
472	VVNNNGQTVFNDILRRGQLLIVPQ	24	2706.4929	110.38	521790000
473	VVNNNGQTVFNDRLRQG	17	1929.9769	93.51	60871000
474	VVNNNGQTVFNDRLRQGQLL	20	2284.2036	188.55	227420000
475	VVNNNGQTVFNDRLRQGQLLIVPQ	24	2721.4674	229.23	840230000
476	VWPGALPGGGVR	12	1164.6404	63.419	34743000
477	VYQPIQTQ	8	975.50255	49.358	798360000
478	WALQRGTSVIPK	12	1354.7721	79.489	47737000
479	WNINAHSVVYMIQ	13	1573.7711	125.97	42379000
480	YDPSEQYQPYPEQQEPF	17	2143.9011	67.08	840310000
481	YDPSEQYQPYPEQQEPFVQ	19	2371.0281	82.85	1596300000
482	YDPSEQYQPYPEQQPF	16	2014.8585	57.39	177310000
483	YFDEQNEQLR	10	1340.5997	138.1	61157000
484	YFGRGPIQIS	10	1136.5978	79.809	4037600000
485	YFGRGPIQISY	11	1299.6612	75.043	0
486	YHNAPALVYIL	11	1272.6867	86.794	109490000
487	YHNAPGLV	8	869.43955	80.377	1567600000
488	YHNAPGLVYLL	11	1258.671	92.187	1354200000
489	YIDRIHKA	8	1014.5611	77.923	34070000
490	YNPSEQYQPYPE	12	1513.6361	72.638	175420000
491	YNPSEQYQPYPEQQEPFVQ	19	2370.0441	73.881	3028000000
492	YQPYPEQQQPILQQ	14	1758.8577	57.204	96959000
